# Effects of Drought on Mortality in Macro Urban Areas of Brazil Between 2000 and 2019

**DOI:** 10.1029/2021GH000534

**Published:** 2022-03-01

**Authors:** C. Salvador, A. M. Vicedo‐Cabrera, R. Libonati, A. Russo, B. N. Garcia, L. B. C. Belem, L. Gimeno, R. Nieto

**Affiliations:** ^1^ Centro de Investigación Mariña Universidade de Vigo Environmental Physics Laboratory (EPhysLab) Ourense Spain; ^2^ Institute of Social and Preventive Medicine (ISPM) University of Bern Bern Switzerland; ^3^ Oeschger Center for Climate Change Research University of Bern Bern Switzerland; ^4^ Departamento de Meteorologia Universidade Federal do Rio de Janeiro Rio de Janeiro Brazil; ^5^ Instituto Dom Luíz (IDL) Faculdade de Ciências Universidade de Lisboa Lisboa Portugal

**Keywords:** Brazil, drought, mortality, age ranges, gender assessment, vulnerability

## Abstract

A significant fraction of Brazil's population has been exposed to drought in recent years, a situation that is expected to worsen in frequency and intensity due to climate change. This constitutes a current key environmental health concern, especially in densely urban areas such as several big cities and suburbs. For the first time, a comprehensive assessment of the short‐term drought effects on weekly non‐external, circulatory, and respiratory mortality was conducted in 13 major Brazilian macro‐urban areas across 2000–2019. We applied quasi‐Poisson regression models adjusted by temperature to explore the association between drought (defined by the Standardized Precipitation‐Evapotranspiration Index) and the different mortality causes by location, sex, and age groups. We next conducted multivariate meta‐analytical models separated by cause and population groups to pool individual estimates. Impact measures were expressed as the attributable fractions among the exposed population, from the relative risks (RRs). Overall, a positive association between drought exposure and mortality was evidenced in the total population, with RRs varying from 1.003 [95% CI: 0.999–1.007] to 1.010 [0.996–1.025] for non‐external mortality related to moderate and extreme drought conditions, from 1.002 [0.997–1.007] to 1.008 [0.991–1.026] for circulatory mortality, and from 1.004 [0.995–1.013] to 1.013 [0.983–1.044] for respiratory mortality. Females, children, and the elderly population were the most affected groups, for whom a robust positive association was found. The study also revealed high heterogeneity between locations. We suggest that policies and action plans should pay special attention to vulnerable populations to promote efficient measures to reduce vulnerability and risks associated with droughts.

## Key Points

- Drought influenced positively non‐external, circulatory, and respiratory mortality in Brazilian urban areas in specific population groups
- Females, children, and the elderly were the populations more at risk
- Drought mostly affected mortality in those locations that suffered higher number and severity of drought conditions

## 1 Introduction

Drought is usually described as a complex phenomenon usually defined as a temporal water availability anomaly, in which the balance between precipitation and evaporation is not sufficient to meet environmental and human water (Vicente‐Serrano et al., 2019; Wilhite & Pulwarty, 2020). It is considered one of the most far‐reaching natural disasters that threaten the health, safety, and livelihood of many populations across the world affecting around 55 million people every year and causing more deaths and human displacements than any other natural hazard (Below et al., 2007; FAO, 2021; UNCCD, 2022; UNDRR, 2021; WHO, 2022; WMO, 2021). Salvador et al. (2020a) developed a comprehensive conceptual diagram displaying the potential pathways in which drought can increase the risk of morbidity and trigger in the worst‐case scenario an increased risk of mortality. Drought can have a multitude of impacts, namely (a) reducing the availability and quality of water, which might lead to a higher risk of dehydration, water‐borne diseases and other infections linked to reduced hygiene; (b) reducing food production and availability, with important nutritional repercussions; (c) exacerbating heatwaves and decreasing air quality, with harmful effects on the respiratory and circulatory systems; and (d) causing severe economic losses and potential forced human migrations, which have been linked to important repercussions in mental health (CDC, 2020, 2021; Grigoletto et al., 2016; Stanke et al., 2013; UNDRR, 2021; Watts et al., 2017). Moreover, the risk of certain vector‐borne diseases, such as dengue, can be promoted by drought episodes, especially in urbanized vulnerable areas due to the establishment of water stagnant conditions and the creation of additional breeding sites for mosquito vectors (Bellizzi et al., 2020; Lowe et al., 2021; Medlock & Leach, 2015). In terms of its driving mechanisms and constraining conditions, drought has been associated with prevailing weather patterns, such as high‐pressure systems and atmospheric blocking conditions (García‐Herrera et al., 2019; Haile et al., 2020), which can contribute to the intensification of warm or cold events (Brunner et al., 2017; Geirinhas et al., 2021; Schaller et al., 2018; Sillmann et al., 2011) with notable implications on mortality (Gasparrini et al., 2015; Geirinhas et al., 2020). Despite the importance of drought in public health, the probability of drought‐related health impacts varies widely and largely depends upon a variety of factors (Stanke et al., 2013), some of them poorly assessed in the relation between drought and specific aspects of health (Salvador et al., 2020a; Sena et al., 2014). This is especially relevant considering the expected increase in the number of people exposed to drought events by the end of the 21st century due to climate change and population growth, which could pose a higher threat to health if adequate mitigation and adaptation measures are not taken (Haines & Ebi, 2019; IPCC, 2021; Watts et al., 2015).

Recent studies have assessed the association between drought exposure and mortality through using different methodological designs and temporal scales in the analyses, suggesting that drought conditions significantly influence the mortality of the population. In Europe, studies done for the Iberian Peninsula revealed that during the last decades the short‐term impact of drought on daily non‐external, circulatory, and respiratory mortality was principally through the effect of heatwaves and air pollution (Salvador et al., 2020c, 2021). Moreover, males and the eldest population were most affected in the Lisbon district (Salvador et al., 2021). In the Western USA, Berman et al. (2017) revealed a significant increase in daily all‐cause mortality risk among older adults during high severity worsening drought conditions and different effects between coastal and interior regions; during worsening drought conditions there was an increased risk of death in interior regions, where this phenomenon occurred less frequently. This fact was also detected by Salvador et al. (2019), who found that interior provinces of northwest Spain (although showing a higher occurrence of drought events) resulted in higher risk of mortality (by non‐external, circulatory and, respiratory causes) compared to coastal provinces. In northern Bangladesh, a significant association between drought severity and monthly mortality was also indicated. Whereas severe and extreme short‐term drought periods mostly affected all‐cause mortality, mild and moderate short‐term drought conditions influenced respiratory deaths. Moreover, both short‐term and long‐term droughts impacted circulatory mortality (Alam et al., 2021). Another recent study conducted across the whole USA (Lynch et al., 2020) reported heterogeneity in the association between drought severity and all causes of death counted per year among adults according to race, climate region, and age, but not sex. However, this study also suggested a possible protective effect of drought. In this aspect, the magnitude of drought impacts is largely determined by socioeconomic variables, including the structure and density of the population (K. R. Smith et al., 2014; UNDRR, 2021). People with low socioeconomic status, agricultural workers in rural areas, children, older adults, and pregnant women are commonly described as the most vulnerable population groups. Therefore, the analysis of the implications of social conditions as modifier factors in the association between drought conditions and health outcomes is a pressing challenge because this phenomenon can affect health through different pathways and disproportionately affect specific groups of the society (Salvador et al., 2020a; Sorensen et al., 2018; UNDRR, 2021). Studies that integrate the gender perspective are essential due to the complexity in the association between gender and vulnerability to drought effects and the heterogeneity observed between regional studies (Lynch et al., 2020; Nellemann et al., 2011; Salvador et al., 2021; Wang et al., 2021; WHO, 2014).

There is growing interest in the implications of drought on both morbidity and mortality, as its intensity and frequency are expected to increase in several regions of the world, including South America (Spinoni et al., 2020, 2021; Watts et al., 2017; Woetzel et al., 2020). Specifically, Brazil is considered vulnerable to current and projected climate extremes (Dai, 2012; IPCC, 2021; Magrin et al., 2007), with a lack of technical and scientific bodywork that can respond to the public health challenges of droughts (Alpino et al., 2016; Sena et al., 2014). Over the last two decades, severe drought episodes have occurred in Amazonia, Cerrado, Pantanal, Northeast and Northwest Brazil (e.g., Jimenez et al., 2021; Libonati et al., 2020; Marengo & Espinoza, 2016; Marengo et al., 2017, 2021; Panisset et al., 2018). In Southeast Brazil, drought conditions have substantially contributed to significant socioeconomic and environmental damages and extreme heatwave events, as a result of pronounced precipitation deficits and blocking atmospheric patterns (Coelho et al., 2016; Geirinhas et al., 2021; Getirana, 2016). The population in Brazil (particularly the semiarid region of Brazil's Northeast) appears to be the most vulnerable to drought exposure and effects associated with climate variability in the present due to low rates of social and economic development (da Silva et al., 2021; Magrin et al., 2007; Menezes et al., 2021; PBMC, 2013; Sena et al., 2014, 2018).

For the first time in Brazil, a comprehensive study is conducted here to assess the short‐term association between drought exposure and weekly non‐external, circulatory, and respiratory mortality between 2000 and 2019, including a stratified analysis according to sex and age ranges of the population. Thus, this study proposes to deepen the understanding of the short‐term impact of droughts on different causes of mortality across Brazil and identify populations at potential risk, providing evidence to facilitate the targeting of actions to reduce vulnerability and risks, and ultimately strengthen the resilience of the Brazilian population.

## 2 Material and Methods

### 2.1 Region of Study: Brazil

**Figure 1:**
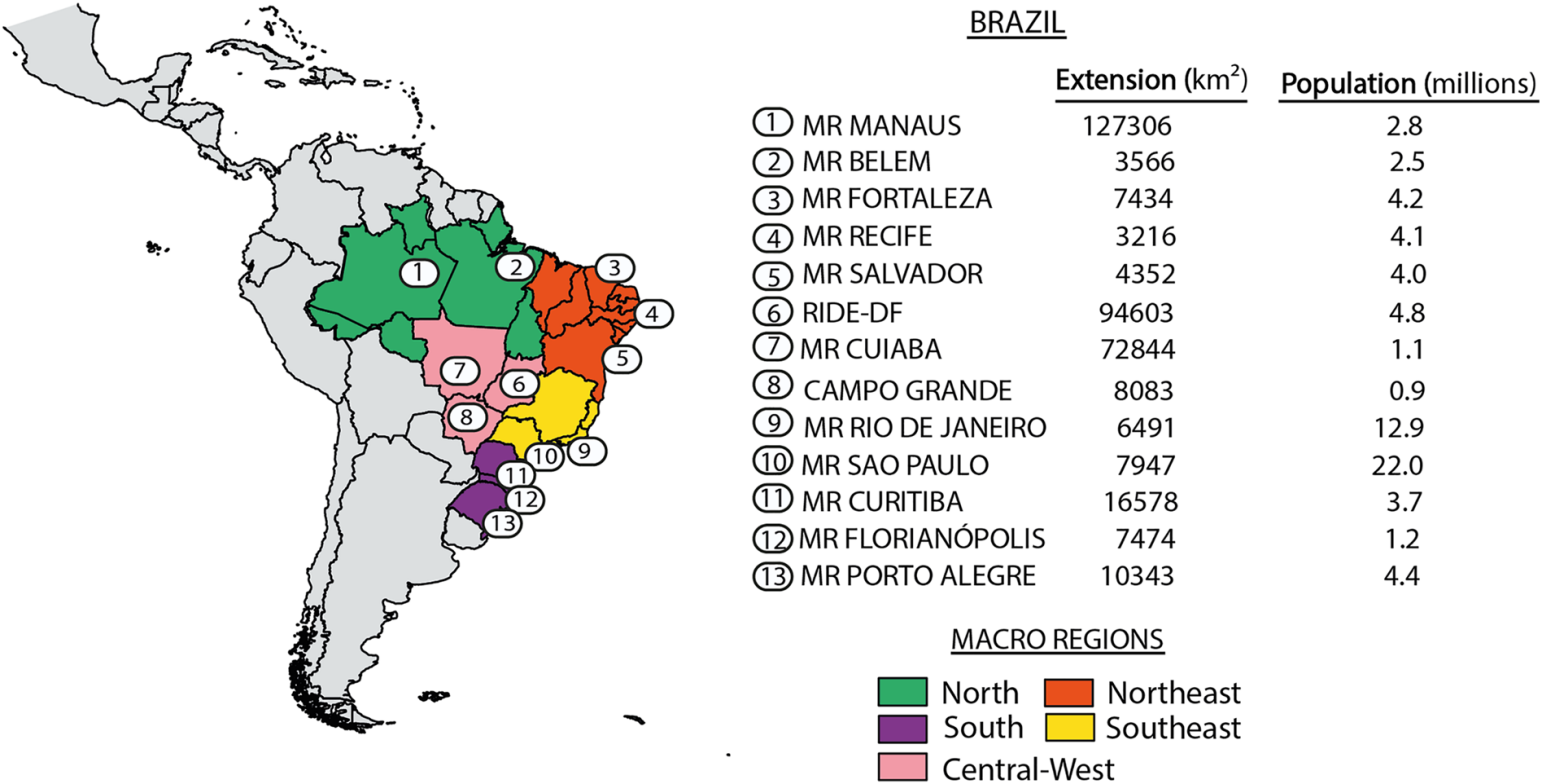
Map of South America representing the study region of Brazil divided into five macro‐regions where the 13 analyzed locations are placed. Each location is denoted with numbers, and their geographical extension is also displayed. The code colors represent the five macro‐regions across the country, namely North (green), Northeast (red), Southeast (yellow), South (purple), and Central‐West (pink). The geographical extension (in km^2^) and the number of inhabitants (in millions, data from IBGE [2021]) are indicated for each location. MR: Metropolitan Region. RIDE‐DF: Development Integrated Region of the Federal District and Surrounding Areas.

Brazil is the largest country in South America composed of 26 states and the Federal District of Brasilia, with a geographical extension of 8,511,965 km^2^ and a population size of almost 213 million inhabitants, being São Paulo the most populated city (12.3 million inhabitants; IBGE, 2021). This study focuses on 13 macro urban areas, which include the 11 principal metropolitan Brazilian regions (MRs), the city of Campo Grande, and the Development Integrated Region of the Federal District and Surrounding Areas (RIDE‐DF) where Brazil's capital (Brasilia) is located (Figure 1). Hereafter we used the term “locations” or “Brazilian locations” to refer to these 13 macro urban areas of Brazil. These areas encompass more than 65 million people which corresponds to one‐third of the country's population. These locations are distributed across the five main macro regions of Brazil, with distinctive climatic characteristics: (a) North (Manaus and Belém), (b) Northeast (Fortaleza, Recife, and Salvador), (c) Southeast (Rio de Janeiro and São Paulo), (d) South (Curitiba, Florianópolis, and Porto Alegre), and (e) Central‐West (Cuiabá, Campo Grande, and RIDE‐DF). According to the updated Koppen's climate classification map for Brazil, the north region is classified as tropical (with a dry season and monsoon); the Northeast is mainly semi‐arid, the Southeast and South are mainly subtropical and the Central‐West is tropical with dry winter (Alvares et al., 2013).

Drought has been described as the disaster that affected the most population in Brazil between 2000 and 2020 (EM‐DAT Atlas), with particular emphasis on the events which took place in the 2010–2016 period in Northeast Brazil (Marengo et al., 2018), in 2014–2015 drought in South‐eastern Brazil (Coelho et al., 2016; Geirinhas et al., 2021), the drought events in 2005, 2010 and 2016 in Amazonia (Jimenez et al., 2018; Jiménez‐Muñoz et al., 2016; Panisset et al., 2018), and in 2019–2020 in Pantanal (Libonati et al., 2020; Marengo et al., 2021; Thielen et al., 2020, 2021). Recently, although outside our study period, the drought of 2021 affecting most South‐eastern and Central‐West has been reported to be facing one of the worst droughts ever recorded (Langenbrunner, 2021), showing the increasing trend toward drier periods.

### 2.2 Study Design and Data

#### 2.2.1 Data Collection

Time‐series data, including mortality (dependent variable), the drought index (independent variable), and mean temperature (confounder) were used for each of the 13 Brazilian locations between 2000 and 2019.

Drought exposure was measured by the Standardized Precipitation‐Evapotranspiration Index (SPEI; Vicente‐Serrano et al., 2010) which is widely used in drought analysis, including studies on health impacts as Lynch et al. (2020) and Salvador et al. (2019, 2020b, 2020c, 2021). Several advantages have been described in comparison to other drought indices for climate studies or to analyze their impacts (Vicente‐Serrano et al., 2012). The SPEI is based on a standard normal variable that can be calculated at different time scales unlike other indices (e.g., Palmer Severity Index), which makes the SPEI comparable in time and space. The calculation procedure of the SPEI is a modification based on the Standardized Precipitation Index (SPI, McKee et al., 1993). Whereas the SPI uses only precipitation data, the SPEI is based on a simple water balance which is determined by the difference between precipitation and evapotranspiration, incorporating the influence of temperature in the drought calculation. Thus, the SPEI is useful to carry out climate variability studies since this index is sensitive to global warming (Vicente‐Serrano et al., 2010). The SPEI time series for each Brazilian location were obtained from the Climatology and Climate Services Laboratory (LCSC) webpage (https://lcsc.csic.es) by the Spanish National Research Council (CSIC). The website provides near real‐time information about drought conditions with a 0.5° spatial resolution and weekly temporal resolution calculated using ERA5 reanalysis data (Hersbach et al., 2020). The SPEI time‐series are available at different time scales (0.5–48 months) from 1979 to the present. In this work SPEI at 1 month of accumulation (SPEI‐1, short‐term) was used, having a value of SPEI‐1 for each week of the drought measure, accounting for 1 month backward in time throughout the whole series (see Vicente‐Serrano et al. [2017] for more details). The SPEI‐1 weekly series were downloaded from 2000 to 2019 from the grill cells where the geographical coordinates of each location are placed, containing the center of each city or metropolitan region. The SPEI can detect both dry and wet conditions, corresponding to negative and positive values of the series, respectively. Following the criteria of Agnew (2000), a drought episode onset when SPEI falls below −0.84, and finishes when it returns to normal or positive values (from −0.84 and above). Moreover, drought events can be classified into categories applying different thresholds to identify the severity of each dry period. Three categories were considered: “moderate” (−1.28 < SPEI ≤ ‐0.84), “severe” (−1.65 < SPEI ≤ −1.28), and “extreme” (SPEI ≤ −1.65) (Agnew, 2000; Stojanovic et al., 2020). SPEI values below −2.33 refer particularly to “exceptional drought,” but here they were accounted for in the descriptive analysis in the extreme category due to the low number of weeks under these conditions. Figure S1 in Supporting Information S1 represents the SPEI‐1 evolution in the RIDE‐DF area across the period 2000–2019, providing an illustrative example to show the continuous weekly series, as well as the different thresholds for each category of severity.

Daily mean temperature data for each Brazilian location were obtained from ERA5 reanalysis (Hersbach et al., 2020) the European Centre for Medium‐range Weather Forecasts (ECMWF). This reanalysis provides hourly worldwide temperature data on a spatial grid of 0.25° × 0.25° resolution, covering the period from 1979 onwards (see https://cds.climate.copernicus.eu/ for more details). The corresponding temperature time series were extracted from the grid cell containing the geographical coordinates of each location. We constructed a weekly series between 2000 and 2019 by averaging the days according to the weekly interval defined by the SPEI. Although we used mean temperature data from ERA5‐reanalysis instead of data from weather stations, evidence indicates that the shape of temperature distribution is very close between both sources (de Schrijver et al., 2021; Royé et al., 2020).

The reason for using the last ERA5 reanalysis is due to its higher confidence verified by several comparisons with other reanalyzes and observations (Hersbach et al. (2020), and references therein); including those accurate for hydrological applications (e.g., Tarek et al., 2020; Xu et al., 2019), as SPEI calculation, and improving on the legacy of its previous version ERA‐Interim data set (Dee et al., 2011; Lorenz & Kunstmann, 2012; Trenberth et al., 2011).

Daily non‐external‐cause mortality (which refers to natural causes without considering external causes such as accidents and violence, injury, and poisoning), as well as daily circulatory‐, and respiratory‐cause mortality data sets were collected by the Unified Health System of Brazil (SUS; https://www.saude.mg.gov.br/sus) and classified according to the 10th Revision of the International Classification of Diseases codes (ICD10: A00‐R99, I00‐I99, J00‐J99, respectively). Mortality databases separated by causes contained information on sex (male and female), age (0–9, 10–44, 45–64, 65–74, ≥75 years), and location.

Since the time resolution of the SPEI‐1 used here is on a weekly scale, we constructed a continuous weekly mortality series adding the daily counts for the interval of days corresponding to each week as they are established for the drought index.

#### 2.2.2 Study Design and Statistical Analysis

A two‐stage time‐series analysis was conducted accounting for multivariate complex associations and pooling methods (meta‐analytical techniques) to estimate the short‐term association between drought events (weekly series of drought measure at 1‐month of accumulation) and weekly mortality in Brazil at the same week of exposure, expressed as relative risk (RR). In addition, impact measures were also estimated through the calculation of the attributable fraction to drought exposure for the 13 macro urban areas as a whole. The analyses were conducted with R software (version 4.0.1) using *dlnm* and *mixmeta* packages.

##### 2.2.2.1 Main Analysis

In this study, quasi‐Poisson regression models were applied in the first stage to estimate the short‐term association between drought events and mortality in each Brazilian location, as in Berman et al. (2017) for the Western USA. This type of model is an alternative to Poisson regression models, which have been used in previous studies conducted in different regions (e.g., Alam et al., 2021; Salvador et al., 2021, 2020c) to deal with the potential overdispersion. Separate models were conducted for each cause of death (non‐external, circulatory, respiratory), sex (total, males, and females), and age groups (all ages; children: 0–9, young people: 10–44; adults: 45–64; older adults: 65–74; the eldest population: ≥75 years old). In the case of circulatory and respiratory mortality, the analysis was conducted only for all ages together due to the low number of cases. We assessed the association between weekly mortality and drought events applying a threshold parametrization in the main model, assuming the effect of the SPEI‐1 values above −0.84 is null, whereas the effect for values below −0.84 is linear. The associations were estimated for the same week's exposure (lag 0). The weekly temporal resolution used (in which the drought index was created) maintains the original fluctuation of the SPEI measurements for each time unit. Seasonal and long‐term trends were controlled in the models with a natural spline of time with two degrees of freedom per year of the study period, and a natural spline of the indicator of the week of the year with three degrees of freedom. All these specifications were selected based on the criteria of quasi‐Akaike information (Pan, 2001). The temperature was also considered as a confounder and included as an explanatory variable in the main models. Distributed Lag Non‐linear Models (DLNM) were used to flexibly account for non‐linear and delayed effects of temperature on mortality (Gasparrini et al., 2010). Following the criteria of quasi‐Akaike information and based on recent studies on the association between temperature exposure and mortality, the temperature exposure‐death relationship was modeled using a natural spline with three internal knots placed at the 10th, 75th, 90th percentiles (*p* = 10th, 75th, 90th) of mean temperature distribution for each location. An unconstrained lag structure up to 3 weeks (lag 0–3) was considered to control the effect of both heat and cold (see the equation of the model in Method S1 of Supporting Information S1).

For each location and population group, short‐term RRs of non‐external, circulatory and respiratory mortality associated with drought events were predicted for each category of drought severity. We established the prediction values at the highest SPEI‐1 values that defined the drought categories (moderate: −1.27; severe: −1.64, and extreme: −2.32).

In the second stage, we pooled area‐specific estimates to derive overall risks using separate meta‐analytical models for each cause of death and population group. From the RRs, we calculated the attributable fractions (AF) to drought separated by each category of severity. These estimations were obtained through the following equation: AF (%): [(RR – 1)/RR] × 100 (Coste & Spira, 1991; Gasparrini & Leone, 2014; Montrull et al., 2016), which represent the percent of deaths attributed to the different conditions of drought severity in the exposed population.

##### 2.2.2.2 Sensitivity and Additional Analyses

Sensitivity analyses were conducted to check the robustness of our findings using data for the total population and non‐external mortality (see Supporting Information S1 for more details). We checked the different combinations for the exposure‐response association of the SPEI‐1 and mean temperature, and we tested different specifications for the seasonality and long‐term trend components.

In the case of the SPEI‐1 variable, we changed the drought onset threshold from −0.84 to 0, and also extended the lag dimension to 3 weeks. We also modeled the exposure‐response association with a natural spline function checking a change in the number and values of the internal knots placing them at the percentiles *p* = 10th, 50th and *p* = 10th, 50th, 90th of the location‐specific SPEI‐1 distributions. Additionally, all these analyses were repeated using a quadratic B‐spline function.

According to the mean temperature, we tested the use of both quadratic B‐spline and natural cubic spline functions with two and three degrees of freedom and also checked different internal knots positions to model the exposure‐response relationship (as in Gasparrini et al. [2015]). Furthermore, we tested both strata and integer functions for the establishment of the lag‐response curve. For seasonal and long‐trend components, we first changed the number of degrees of freedom in the time indicator ranging from 4 to 7, whereas we also tested to change the number of degrees of freedom in the time indicator varying from 2 to 4 and incorporating an indicator of the week of the year, as a linear term and then using a natural spline with 2 and 3 degrees of freedom.

The modeling choices were evaluated through the criterion of quasi‐Akaike information (Table S1 in Supporting Information S1), and a comparison of an overall association between drought and non‐external mortality was conducted using the following best‐fit model varying the fit of the seasonal and long‐term trend (Table S2 in Supporting Information S1). We performed additional sub‐analysis to display the overall association between drought exposure and non‐external mortality risk exploring more complex approaches, such as establishing a different threshold to define the drought onset (SPEI‐1 = 0) and exploring the potential non‐linearity (Figure S2 in Supporting Information S1).

Another additional analysis was conducted to assess the interaction between drought conditions and mean temperature (Table S3 in Supporting Information S1). In this pathway, we defined the drought index as a dichotomic variable (0 = “no drought,” and 1 = “drought” considering the threshold in −0.84 value of the SPEI‐1), and the mean temperature variable was included as in the main model.

## 3 Results

### 3.1 Descriptive Analysis

Table 1 summarizes the descriptive statistics on total counts of non‐external, circulatory, and respiratory mortality, drought conditions measured by the weekly SPEI‐1, and the averaged weekly mean temperature for the analyzed locations between 2000 and 2019. Figure S3 in Supporting Information S1 shows the scatter plot of the weekly non‐external mortality in the total population and the weekly SPEI‐1 series for each analyzed location. Table S4 in Supporting Information S1 shows the total counts for the different causes of mortality segregated by sex and age ranges. A total of 6,558,839 non‐external deaths (3,376,523 males and 3,182,316 females) were analyzed in the selected locations across the study period, with circulatory mortality accounting for 32.94% and respiratory mortality for 13.06% (Table 1, Table S4 in Supporting Information S1). The highest number of deaths for these three groups of causes were recorded in São Paulo, Rio de Janeiro, and Porto Alegre, in contrast to Campo Grande, Cuiabá, and Florianópolis. According to age ranges, 4.69% of the total non‐external deaths corresponded to children (0–9 years), 9.50% to young people (10–44 years), 26.83% to adults (45–64 years), 20.65% to older adults (65–74 years), and 38.33% to the eldest population (≥75 years).

**Table 1:** Descriptive Statistics of Total Mortality Data (From the Unified Health System of Brazil (SUS)) and Climatic Variables (SPEI‐1 From the Climatology and Climate Services Laboratory, CSIC, Spain; and Mean Temperature From ERA5) for Each Brazilian Location and for the Whole of Them Between 2000 and 2019

Regions	Mortality causes			Climatic conditions (weekly temporal resolution)				
	No‐external	Circulatory	Respiratory	Weeks with drought conditions	Weeks with moderate drought	Weeks with severe drought	Weeks with extreme drought	Mean temperature (range) (in °C)
MR Manaus	164,123	33,985	15,740	219 (22.8%)	107 (48.9%)	64 (29.2%)	48 (21.9%)	26.8 (24.0–31.6)
MR Belém	202,455	55,291	29,590	199 (20.7%)	105 (52.8%)	70 (35.2%)	24 (12.1%)	24.2 (25.0–28.9)
MR Fortaleza	319,439	90,565	39,645	220 (22.9%)	118 (53.6%)	61 (27.7%)	41 (18.6%)	26.9 (24.9–28.5)
MR Recife	413,415	144,760	57,472	211 (22%)	110 (52.1%)	60 (28.4%)	41 (19.4%)	24.8 (21.9–27.5)
MR Salvador	320,557	100,405	37,851	211 (22%)	101 (47.9%)	73 (34.6%)	37 (17.5%)	25.1 (20.4–29.7)
RIDE‐DF	269,597	85,760	27,377	288 (30%)	148 (51.4%)	85 (29.5%)	55 (19.1%)	22.4 (16.8–29.0)
MR Cuiabá	86,770	27,353	10,145	301 (31.4%)	159 (52.8%)	96 (31.9%)	46 (15.3%)	26.2 (17.0–21.2)
Campo Grande	77,312	26,939	16,605	264 (27.5%)	120 (45.5%)	96 (36.4%)	48 (18.2%)	24.5 (13.3–30.6)
MR Rio de Janeiro	1,682,979	539,703	214,108	249 (25.9%)	128 (51.4%)	54 (21.7%)	67 (26.9%)	21.6 (14.2–27.5)
MR São Paulo	2,111,312	763,774	293,136	249 (25.9%)	123 (49.4%)	72 (28.9%)	54 (21.7%)	20.0 (11.1–26.7)
MR Curitiba	312,112	102,516	37,306	228 (23.8%)	127 (55.7%)	71 (31.1%)	30 (13.2%)	17.8 (7.0–25.4)
MR Florianópolis	88,393	31,204	10,409	196 (20.4%)	97 (49.5%)	64 (32.7%)	35 (17.9%)	18.1 (6.5–27.0)
MR Porto Alegre	510,375	158,446	66,909	193 (20.1%)	99 (51.3%)	58 (30.1%)	36 (18.7%)	19.7 (7.8–30.2)
Total	6,558,839	2,160,701	856,293	233 (24.3%) (average)	119 (51.1%) (average)	96 (41.2%) (average)	46 (19.7%) (average)	23.2 (6.5–31.7) (average)

*Note.* Records of the total number of non‐external, circulatory, and respiratory deaths are indicated. Total weeks with drought conditions (the number and percentage of the total weeks during the study period) and separated by severity levels (including the number of weeks in total and the percentage within the count of weeks under drought conditions) are also shown. Information on the mean and its range of the temperature (in °C) is also included.

In terms of drought exposure, along the study period 2000–2019, the results show that the percentage of weeks with short‐term drought conditions ranged from 20.1% to 31.4% comparing all the locations (Table 1). Moderate drought conditions were the most common, followed by severe conditions, with the extreme droughts being the least frequent, as expected. The results (Table S5 in Supporting Information S1) show that the year 2012 accounted for the highest number of weeks with drought for all the locations together (236 weeks). This maximum is due to the great weight of the drought that occurred in the semiarid Northeast region, which has been experiencing a large‐scale drought since 2010 (Jimenez et al., 2021; Marengo et al., 2018). However, this area was not the one with the highest percentage of droughts (Table 1), which were achieved in the Central‐West (Cuiabá: 31.4%, RIDE‐DF: 30%, and Campo Grande: 27.5%). At the opposite extreme are the global lowest values recorded in the North (particularly in Belém: 20.7%) and in the South (Florianópolis: 20.4% and Porto Alegre: 20.1%).

This heterogeneity between regions was also observed when drought was categorized by severity, where the Southeast and Central‐West (Rio de Janeiro, São Paulo, and RIDE‐DF) regions had the highest number of weeks categorized as extreme drought.

It is important to notice that the variability in the drought exposure along the last two decades becomes more evident when each region (or macro‐region) is analyzed individually over time. Due to the vast continental extension of Brazil, it has experienced droughts in distinct areas and not always at the same moment. Our results show that over the Central‐Western region faced the higher weeks of drought conditions during 2010 (mainly over the western region of Cuiabá, as Andreoli et al. [2016]) reported), following by 2007 (when RIDE/DF was more affected, see de Araújo et al. [2012]), and by 2019 (mainly over the southern portion Campo Grande; this last event follows in 2020 (out of this study as shown Libonati et al. [2020] and Marengo et al. [2021]). In the Southeast, unprecedented drought conditions occurred during 2014, being more severe during the austral summer seasons of 2013/14 and 2014/15 (see Coelho et al., 2016; Geirinhas et al., 2021; Getirana, 2016). The vast Amazon region (in the Northern region) was affected by several drought periods, but each event presented very different temporal and spatial patterns. The event of 2015 presented its epicenter in the eastern portion, while in 2005 mainly occurred over the western Amazon region, whereas the 2010 event took place in the southwestern and south‐eastern parts (Marengo & Espinoza, 2016; Panisset et al., 2018).

### 3.2 Short‐Term Effects of Drought Severity Levels on Non‐External, Circulatory, and Respiratory Mortality

**Figure 2:**
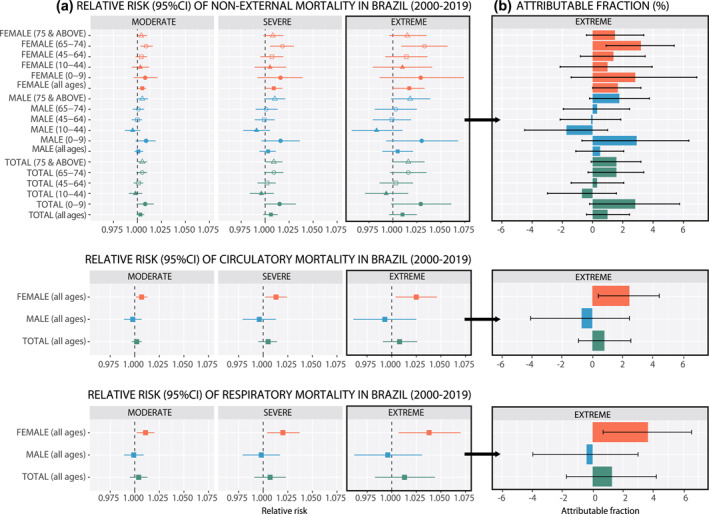
(a) The overall short‐term association between drought and non‐external mortality expressed in relative risk and 95% confidence intervals per each category of drought severity measured by the Standardized Precipitation‐Evapotranspiration Index calculated at 1 month of accumulation (SPEI1) in Brazil from 2000 to 2019. (b) The attributable fractions among the exposed population and 95% confidence intervals (in percentage) to extreme drought for the different causes of death and population groups.

Figure 2a illustrates the overall short‐term association (RRs and 95% confidence intervals [95%CI]) between each category of drought severity and mortality cause for each group of sex and age. Table S2 in Supporting Information S1 includes the complete set of statistics, including the RR values, 95% confidence intervals, and the AF for Brazil overall. According to Figure 2a, drought events were associated with an increase in non‐external mortality risk in all analyzed population groups, except in people aged 10–44 years old in the total population and males. For the total population, the RRs varied from 1.003 [0.999–1.007], 1.006 [0.998–1.013], and 1.010 [0.996–1.025] associated with moderate, severe, and extreme drought conditions, respectively. Hereafter the RRs [95% CI] are always listed in the same order as in this last case for the three drought categories. Larger risks were observed in females (1.005 [1.000–1.009], 1.009 [1.000–1.018], and 1.017 [1.000–1.033]), compared to males (1.001 [0.997–1.006], 1.003 [0.994–1.011], and 1.005 [0.989–1.021]). Also, larger risks were observed in the total population for children (RRs varied from 1.008 [0.999–1.017], 1.015 [0.999–1.032], and 1.029 [0.998–1.061]), and the eldest group (≥75) with RRs of 1.005 [1.000–1.010], 1.009 [1.000–1.018], and 1.016 [0.999–1.033]; similar values were found for 65–74 group. Particularly, non‐external mortality risks in females aged 65–74 years old ranged from 1.009 [1.003–1.016] to 1.033 [1.009–1.057] associated with moderate and extreme drought conditions; in the case of men aged ≥75 the risks varied from 1.005 [0.999–1.011] to 1.018 [0.998–1.039]. For the remaining age ranges, our results showed a positive association (but lower and imprecise) in the 45–64 group among the total population and females, whereas a null effect was estimated in males. The results for the 10–44 group were not consistent comparing total, females, and males.

According to specific causes of death, Figure 2a shows a positive association between both circulatory and respiratory mortality risks and drought events in total and females, whereas a negligible negative effect was observed in the case of males. The association was mostly observed in females in both causes, with a slightly higher risk for respiratory mortality (1.038 [1.007–1.070]) than for circulatory mortality (1.025 [1.004–1.046]) associated with extreme drought.

Figure 2b displays the AF to extreme drought for each cause of death among the exposed population, which represents the highest proportion of deaths attributable to short‐term drought conditions according to the highest severity threshold defined in this study (SPEI‐1 = −2.32). The AF corresponding to the remaining categories of drought severity are shown in Table S2 of Supporting Information S1. The percentage of non‐external deaths attributable to extreme drought events was 0.99% in the total population, 2.82% in children, and 1.57% both in the total population aged 65–74 and ≥75. Attributable mortality to extreme drought in females was 1.67%, whereas in males 0.50%. Moreover, a larger fraction was found in females aged 65–74 years (3.19%). Attributable mortality for circulatory causes in the total population was 0.79% and for respiratory causes 1.28%. Larger AF were also observed in females for both circulatory (2.44%) and respiratory (3.66%) causes. However, in males there was a mild reduction in the proportion of deaths attributable to extreme drought conditions of 0.70% for circulatory mortality and 0.40% for respiratory mortality.

**Figure 3:**
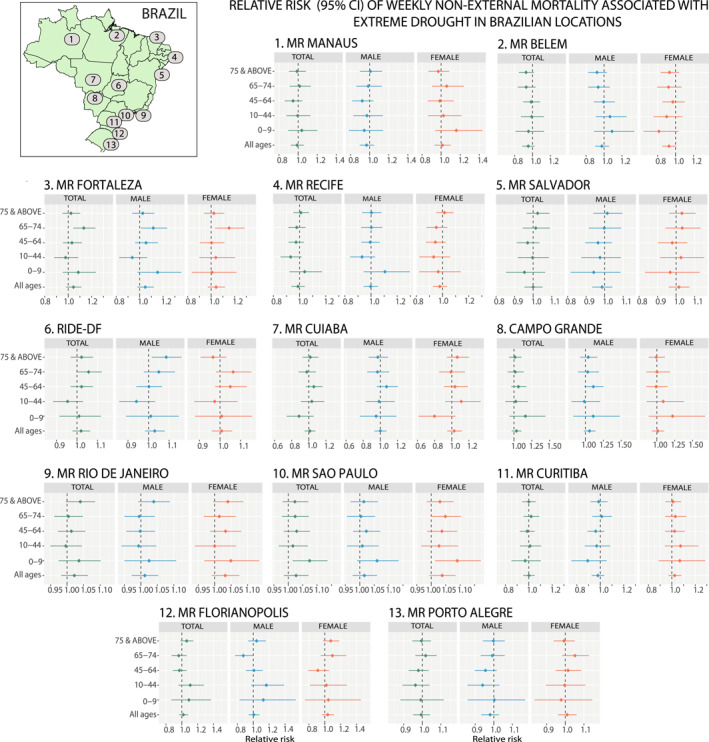
Specific association between extreme drought conditions and non‐external mortality in terms of relative risks and the 95% confidence intervals across the population separated by sex groups and age ranges in each Brazilian location between 2000 and 2019. Drought events were measured by the Standardized Precipitation‐Evapotranspiration Index obtained at one month of accumulation (SPEI‐1).

The estimation of RRs and 95% confidence intervals of non‐external mortality associated with extreme drought conditions in each Brazilian location during the study period is represented in Figure 3, to report the highest risk values related to drought events across the total population, males, and females according to the highest severity threshold defined in this study. Tables S6–S8 in Supporting Information S1 show the results for each category of drought severity. According to our results, there was great heterogeneity in the association between drought events and non‐external mortality risk between regions, and not all population groups were equally affected. Evidence of a positive association was mostly observed in those urban areas located in the Central‐West (RIDE‐FD), Northwest (Fortaleza), and in the Southeast (São Paulo and Rio de Janeiro) for specific groups of the population. In general, Fortaleza and São Paulo were the regions most affected. It is worth noting that the groups at more risk were children in São Paulo (1.074 [1.015–1.136]) and the elderly (Figure S4, Tables S6–S8 in Supporting Information S1). For instance, in Fortaleza, the RRs of non‐external mortality associated with extreme drought in older adults aged 65–74 years reached up 1.133 [1.043–1.232], 1.116 [1.010–1.234], and 1.153 [1.034–1.286] in the total population, males, and females, respectively. On the other hand, and surprisingly, a clear reduction in non‐external mortality associated with drought events was also found in Florianópolis in older male adults (65–74 years old).

Table S9 in Supporting Information S1 shows the risk estimates for circulatory and respiratory mortality of the total population, males, and females (all ages) associated with the different categories of drought severity for each Brazilian location. The main results suggested high variability between locations and the association between drought conditions and specific causes of death differed between the total, males, and females. For instance, drought contributed positively to a higher risk of circulatory mortality in females in Curitiba (with RRs that grows from 1.022 [1.000–1.044] for moderate to 1.076 [0.999–1.159] for extreme conditions), whereas in the case of RIDE‐DF it was observed in the total population and males (RRs varied from 1.015 [1.000–1.031] to 1.053 [0.999–1.11] in the first group, and from 1.023 [1.005–1.042] to 1.083 [1.019–1.151] in the later, respectively). However, a negative association between drought exposure and circulatory mortality was also found in Salvador for the male population (RRs varied from 0.951 [0.924–0.978] to 0.84 [0.763–0.926] for moderate to extreme drought events). Regarding respiratory‐cause mortality, a positive association was detected in São Paulo in the total population, males, and females, with a higher risk in the case of females (RRs reached up 1.071 [1.017–1.128] in the total population, 1.087 [1.025–1.152] in females, 1.057 [1.001–1.116] in males, during extreme drought conditions). However, a clear reduction in the risk of respiratory mortality associated with drought was estimated in Belém for the total population and men (RRs varied from 0.946 [0.91–0.984] to 0.827 [0.723–0.945] in the first group, and from 0.935 [0.891–0.982] to 0.794 [0.672–0.939] in the later). For the rest of the locations, evidence of a robust association between short‐term drought conditions and specific mortality causes was not found.

Sensitivity analysis and additional tests were conducted for overall Brazil to validate the results in the time‐series analysis. According to Figure S4 in Supporting Information S1, a much lower and imprecise association between drought exposure and any cause of death was observed considering up to 3 weeks of delay in comparison with the main analysis without considering lags. In addition, the sensitivity analysis evidenced stability and robustness in the results obtained since the modification of the model did not alter the patterns observed (Table S2 in Supporting Information S1). On the other hand, Table S3 in Supporting Information S1 showed that the impact of drought measured by the SPEI‐1 on non‐external mortality was not substantially modified by the temperature effect in any location.

## 4 Discussion

This epidemiological study reports for the first time the short‐term association between different levels of drought severity and non‐external, circulatory, and respiratory mortality across 13 major urban areas in Brazil during the first two decades of the 21st century. The analysis was adjusted by temperature, and a stratified assessment was included according to sex and age groups to determine the structure of the population at risk in these areas in Brazil. Overall, the main findings suggested a positive association between drought severity and different causes of mortality in the total population, with robust associations for specific population groups.

Accounting for all Brazilian locations as a whole, the risk estimates (RR [95% CI]) in the total population associated with moderate, severe, and extreme drought conditions varied from (a) 1.003 [0.999–1.007], 1.006 [0.998–1.013], 1.010 [0.996–1.025] for non‐external mortality, respectively, (b) 1.002 [0.997–1.007], 1.005 [0.995–1.014], and 1.008 [0.991–1.026] for circulatory mortality, and (c) 1.004 [0.995–1.013], 1.007 [0.991–1.023], and 1.013 [0.983–1.044] for respiratory mortality. The low number of studies on the effects of drought on mortality make the comparison between studies limited. This difficulty is further compounded by the fact that the small number of published studies exhibits differences between them in terms of study design, modeling approach, and definition of the exposure variable (e.g., variability in the type of drought index and its incorporation as a continuous or categorized variable, the time scale used, the criteria for establishing drought conditions, etc.) which may influence the magnitude of the drought‐mortality risk association. Findings from recent studies carried out in regions as far apart as Bangladesh and the Iberian Peninsula revealed a significant influence of drought events in all causes of mortality as well as in non‐external, circulatory, and respiratory deaths in the total population, respectively (Alam et al., 2021; Salvador et al., 2020c, 2021). In this work, the results obtained here are in line with the ones mentioned when stratified analyses were conducted.

Our overall results accounting for the 13 Brazilian macro urban areas suggested strong gender differences, with a robust positive association in females (e.g., RRs were 1.017 [1.000–1.033] for non‐external mortality, 1.025 [1.004–1.046] for circulatory mortality, and 1.038 [1.007–1.070] for respiratory mortality, related to extreme drought). In addition, disaggregating by age, we also found a strong association in children (RRs for non‐external mortality varied from 1.008 [0.999–1.017] to 1.029 [0.998–1.061] related to moderate and extreme drought), and older adults (e.g., RRs for non‐external mortality were 1.016 [0.999–1.033] in the eldest total population, and 1.033 [1.009–1.057] in females aged 65–74 years, related to extreme drought). However, the association between drought and mortality was imprecise for the male group, as well as for the young and young adults for the total population and both sex groups. Other studies across different regions also have indicated that the effects of drought on mortality are dependent on drought severity, with higher implications mostly associated with higher severity levels (Alam et al., 2021; Berman et al., 2017; Salvador et al., 2019; Stanke et al., 2013), which is in accordance with our results. Our findings also support the conclusions of several studies that highlighted that drought can impact human health through different mechanisms, mostly affecting vulnerable populations as children under 5 years, the elderly, and females (Algur et al., 2021; Alpino et al., 2016; Salvador et al., 2020a; Stanke et al., 2013; UNDRR, 2021; Yusa et al., 2015). In terms of health impact, we found that the excess mortality risk attributable to extreme drought exposure in the total population was 0.99%, exceeding that observed to heat in Brazil (0.70%) (Gasparrini et al., 2015). For specific population subgroups, the percentage grows up to 2.82% in children, to 1.57% in the elderly, and to 1.67% in the whole female group, reaching 3.19% in females aged 65–74 years.

Short‐term effects of drought are commonly related to water scarcity and contamination (both chemical and microbiological), and consequent food shortages. These effects can lead to a higher risk of dehydration, food insecurity, and a higher incidence of diarrhea, constituting major causes of infant mortality (Ole‐MoiYoi, 2013). Lack of water associated with drought can threaten sanitation and hygiene and disrupt health services with notable implications on public health (Grigoletto et al., 2016; WMO, 2022). In addition, drought can worse both respiratory health, as was found in studies conducted in China and Brazil (Machado‐Silva et al., 2020; Wang et al., 2021), and circulatory health (Bell et al., 2018; CDC, 2021; UNDRR, 2021), and ultimately trigger an increased risk of mortality by these causes (Salvador et al., 2020c, 2021). In this context, evidence indicates that drought is an important driver of air quality increasing dust and particulate pollution (K. R. Smith et al., 2014; UNDRR, 2021; Vicente‐Serrano et al., 2020; Watts et al., 2017), which have significant impacts on non‐external, circulatory, and respiratory causes of death (Díaz & Linares, 2018; Salvador et al., 2019, 2020a).

Children are generally more vulnerable to potential effects of drought such as decreased air quality or restriction of food supplies due to their physiological susceptibility, immature immune system, and higher exposure because they are frequently involved in vigorous activities spending longer periods of time outdoors, and have higher minute ventilation (Buka et al., 2006; Ebi & Paulson, 2007; K. R. Smith et al., 2014). Particularly in the Brazilian Amazon drought exposure has been reported to worsen respiratory health in children under 5 years old during the dry season, aerosols being the primary driver of hospitalizations (L. T. Smith et al., 2014). The elderly population is also especially vulnerable to climatic and environmental effects on health due to their weakened immune system, low mobility, high prevalence of chronic disease and comorbidities, and lower capacity of restoration of homeostasis. Older people have a higher risk of dehydration than young people due to physiological changes associated with the aging process. In addition, they usually have lower resources or live isolates, which increases their susceptibility (Harper, 2019; NASEM, 2020; K. R. Smith et al., 2014). In accordance with our results, Berman et al. (2017) and Salvador et al. (2020b) highlighted the especial vulnerability of the aging population in the drought risks on mortality in comparison to young adults (Salvador et al., 2021). In line with the results obtained in this study by sex groups, several studies, including the recent special report on drought from the United Nations Office for Disaster Risk Reduction (UNDRR, 2021), have indicated that females are generally more vulnerable to the health effects of drought. This is because they usually face additional risks associated primarily with gender inequalities and gender roles, such as differences in the control over or access to resources and educational or employment status and opportunities between males and females, among others (Desai & Zhang, 2021; UNDRR, 2021). In addition, these inequalities may result in different health outcomes in different countries or communities, due to religious, ethnic, or social norms (Algur et al., 2021; Cardona et al., 2012). Particularly in Brazil, it has been reported that females have higher experiences of discrimination than males (Macinko et al., 2012), as well as worse health status and a greater demand and use of health services that have been commonly associated with gender behaviors, socially and culturally constructed (e.g., repercussions due to work overload in females related to additional responsibilities relative to homework and care), which could influence in higher vulnerability (Aquino et al., 1992; Cobo et al., 2021). All these factors make the association between drought exposure and vulnerability between males and females complex, with pre‐existing regional and cultural differences. Even for two developed countries the results found in the mortality‐drought relationship are different, as shown for example, by a recent study conducted in Lisbon (Portugal) which suggested that males were more affected than females by short‐term droughts in terms of daily mortality (Salvador et al., 2021). However, Lynch et al. (2020) revealed that drought did not affect males and females differently on all‐cause mortality among adults in the USA. Therefore, we firmly believe that further and conclusive studies are needed to clarify the association between drought exposure and mortality between both sex groups. This is particularly important given that climate change and the expected increase in drought frequency and intensity in several parts of the world, including South America, could act as intensifying factors of existing structural social inequalities, affecting disproportionately disadvantageous and susceptible populations (Watts et al., 2020). In addition, projected rapid population growth and aging could imply additional care and economic burdens in the future.

On the other hand, the present study also reported heterogeneity in the association between drought conditions and non‐external, circulatory, and respiratory mortality risks across the analyzed population groups between the 13 Brazilian locations. Similar studies conducted in Spain (Salvador et al., 2020b, 2020c), Bangladesh (Alam et al., 2021), and the United States (Lynch et al., 2020) also revealed regional differences in the effects of drought on mortality within the countries themselves. The magnitude of the risk is defined by the interrelation between the hazard characteristics, exposure degree, and vulnerability of the population. Thereby, different variables could influence the differences observed between the analyzed Brazilian regions. For instance, number of weeks under drought conditions and severity level, local environment and urbanization rates, population structure and density, differences in water access and use trends, environmental awareness and degradation, human welfare, as well as social and economic development, including differences in preparedness and adaptation degree (e.g., higher health structures, health care capacity, socio‐economic status of the population) (Ebi & Bowen, 2016; Miyan, 2015; Naumann et al., 2014; UNDRR, 2021). We found a clear positive short‐term association between drought exposure and the risk of different mortality causes in specific population groups, mainly in those areas that suffered higher occurrence of weeks under drought conditions and that were categorized with the highest level of severity (in line with the results by Salvador et al. (2019) for the northwest Iberian Peninsula). These locations were RIDE‐DF (Central‐West), Fortaleza (Northwest), and Rio de Janeiro and São Paulo (Southeast), being Fortaleza and São Paulo especially vulnerable to drought effects on mortality. Moreover, both children (in São Paulo) and the elderly population were the more at‐risk groups at a regional level. In this context, it has been described that the semiarid Northeast region of Brazil is characterized by low socioeconomic development and low levels of health services (Menezes et al., 2021; Sena et al., 2014), whereas the midwest part presents medium levels, with the south and southeast parts being the areas with the highest (Albuquerque et al., 2017). However, the fact that São Paulo is the most densely populated urban area may make this territory particularly vulnerable to the effects of drought (MAEUEC, 2021; UNDRR, 2021). In contrast, our results also suggested a negative association between drought and mortality risk in regions that suffered fewer weeks of drought and extreme conditions such as Belém (in the North) or Florianópolis (in the South). The negative associations observed between drought and mortality for specific population groups in some Brazilian locations could be associated with the fact that drought may have a protective effect on mortality such as the reduction in flooding in vulnerable populations to such phenomenon, in the same way that Lynch et al. (2020) speculated for the USA.

Evidence of a fluctuation in the risk of the different analyzed causes was observed between both sex groups across the different Brazilian locations, showing inconsistent results. In some locations drought mainly influenced mortality in males while in others it was in females. We argue that social variables could have principally explained these differences. For instance, male populations in certain locations, including outdoors workers such as farmers, or those who engage in doing exercise or activities outside for long periods of time, might have been more exposed to drought. Different access to social services and activities focused on providing support and strengthening resilience in vulnerable gender populations could have influenced differences in mortality risks associated with drought between males and females in different regions. However, a more exhaustive assessment is required to obtain a better understanding of the factors that might have influenced mortality risks related to drought exposure between both sex groups at a regional level.

This ecological time‐series study has some advantages that should be highlighted. The short‐term association between drought exposure and mortality was evaluated for all major urban and highly populated areas of Brazil covering a large study period of 20 years. Impact measures were estimated for different levels of drought severity, mortality causes, and population groups according by sex and age ranges, which has important implications for public health. Another strength is the use of robust statistical models that account for overdispersion of the data and that were adjusted by temperature. The best fit model was chosen following the criteria of quasi‐Akaike information. Moreover, sensitivity analysis was carried out to confirm the consistency of our results, and additional tests also provided additional information to the study on the association between drought exposure and mortality.

In addition, we have used the SPEI to monitor the drought, the index widely used in the scientific literature with undisputed advantages in comparison with other drought indices (Beguería et al., 2014). The availability of this gridded index at a weekly time resolution, instead of monthly, allows obtaining finer estimates on the association between drought exposure and health outcomes. Applying a weekly rather than monthly model allows a better control of the acute confounding factor (temperature). Moreover, the ERA5 reanalysis data was used to calculate the SPEI and the weekly mean temperature, being the most recent and improved climate global‐scale gridded data set from the ECMWF. Furthermore, it has been shown that these data are equally effective in the calculation of mortality risks as those from weather station data (Derouin, 2021).

However, some limitations of this study should be noted. First, although this study included the main Brazilian metropolitan regions with different climatic and social characteristics, our estimates should not be interpreted nationwide as rural regions could not be included in the analysis (which have been described as especially susceptible to drought occurrence, with less investment and public health services compared to the most urbanized areas (Albuquerque et al., 2017). Stratified analysis by age groups could not be assessed for circulatory and respiratory mortality due to the lower number of cases to conduct a powerful statistical analysis. In addition, the control of the effect of atmospheric pollution in the main analysis could not be included, which is one of the known environmental effects on human health during some atmospheric configurations associated with droughts. Although the seasonal and long‐term trends were controlled in the main analysis, some limitations linked to any ecological study should be considered, such as the results cannot be extrapolated at an individual level and that climatic variables do not represent an individual exposure (Barceló et al., 2016; Gelfand, 2010). Thereby, this study is used to estimate associations between exposure and outcome, but not causal links (Levin, 2003).

In view of the differences obtained in this study for different age groups, sex and regions, a deeper understanding of the association between drought and mortality risks would require future analysis to include the implications of other socio‐economic variables as modifier factors in this relationship to minimize risks and protect the health of the population. Furthermore, other specific causes of mortality and morbidity such as those due to lack of water quantity and quality (e.g., intestinal infectious diseases), nutritional and metabolic diseases, or mental disorders, can be also addressed in the future using drought measures at different time scales. In this context, the control of other extreme climatic phenomena strongly linked to drought such as wildfires and the estimations of health impacts from an integrative point of view is also recommended.

## 5 Conclusions

Evidence of an association between drought exposure and non‐external, circulatory, and respiratory mortality was mostly observed in specific population groups for 13 urban areas in Brazil. Overall, drought effects on mortality increased as the drought severity increased in the total population. Females, children, and the elderly were the populations more at risk. Furthermore, heterogeneity in the mortality risks associated with drought conditions was observed across the different population groups. Those regions which suffered higher occurrence of weeks under drought conditions and categorized by the highest severity level such as São Paulo, Fortaleza, RIDE‐DF and Rio de Janeiro were most affected, where drought increased the risk of mortality mostly in children (only in São Paulo) and in people aged 65–74 and 75 and above. However, a protective effect of drought was also found in specific population groups in Florianópolis, Salvador, and Belém. Additionally, females and males were not equally affected across the 13 urban areas in Brazil, which bring us to the fact that it would be vital to carry out additional analysis to obtain a higher understanding of those factors that could influence these differences. Based on the main findings of this study, public health policies and strategies must give special attention to vulnerable groups of the population to implement more effective actions to reduce vulnerability and risks, and ultimately strengthen the population resilience. This is especially important considering the expected increase in frequency and intensity of drought events in several regions such as South America in the future, which may interact with and increase the social inequalities, intensifying drought effects on health in the most disadvantaged populations.

## Conflict of Interest

The authors declare no conflicts of interest relevant to this study.

## Supporting information

Supporting Information S1

## Data Availability

Weekly mortality data were obtained from official daily death certificates of the Brazilian Health System database DATASUS, 2021 [Data set]. https://datasus.saude.gov.br/transferencia-de-arquivos/, which are publicly available through the Data Science Platform developed by the Institute of Scientific and Technological Information in Health of the Oswaldo Cruz Foundation. Weekly SPEI‐1 series were freely downloaded from the LCSC (Climatology and Climate Services Laboratory), 2021 [Data set]. https://global-drought-crops.csic.es/#map_name=all_spei_1#map_position=4. Daily temperature series were obtained from the European Centre for Medium‐Range Weather Forecasts public datasets (ECMWF), 2021 [Data set]. https://cds.climate.copernicus.eu/cdsapp#!/dataset/reanalysis-era5-single-levels?tab=form. Data set supporting the conclusions of this research and the R code used in the main analysis can be found in Mendeley data repository. Files are attached in Salvador et al. (2022). Effects of Drought on Mortality in Macro Urban Areas of Brazil between 2000 and 2019: Data set [Data set]. Mendeley Data, V1. http://dx.doi.org/10.17632/bbj27mxx9g.1.
