# Supplementary material for: Effects of Drought on Mortality in Macro Urban Areas of Brazil Between 2000 and 2019
Source: Geohealth. 2022 Mar 1;6(3):e2021GH000534. doi: 10.1029/2021GH000534 (PMC8902811; doi:10.1029/2021GH000534)
Supplement: Supplementary file 1 — Supporting Information S1 [file GH2-6-e2021GH000534-s001.doc]

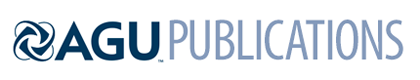


*GeoHealth*

Supporting Information for

***Effects of Drought on Mortality in Macro Urban Areas of Brazil***

***between 2000 to 2019***

C. Salvador^1,2,3^, A.M. Vicedo-Cabrera^2,3^, R. Libonati^4,5^, A. Russo^5^, B.N. Garcia^4^, L.B.C. Belem^4^, L. Gimeno^1^, R. Nieto^1^

*^1^ Centro de Investigación Mariña, Universidade de Vigo, Environmental Physics Laboratory (EPhysLab), Ourense, Spain*

*^2^  Institute of Social and Preventive Medicine (ISPM), University of Bern, Switzerland*

*^3^ Oeschger Center for Climate Change Research, University of Bern, Switzerland*

*^4^ Departamento de Meteorologia, Universidade Federal do Rio de Janeiro, Rio de Janeiro, Brazil*

*^5^ Instituto Dom Luiz (IDL), Facultade de Ciências, Universidade de Lisboa, 1749-016, Lisbon, Portugal*

Corresponding author:

e-mail: coral.salvador@ispm.unibe.ch (C.Salvador)

**DATA AND ACKNOWLEDGMENTS**

- This study was generated using daily non-external-, circulatory-, and respiratory- cause mortality datasets, segregated by sex and age groups, from the Unified Health System of Brazil (SUS) [ICD10 codes: A00-R99, I00-I99, J00-J99, respectively]. These databases were processed to obtain weekly time series for each cause of death for each subgroup of the population (total, males, females, and age ranges).
- Drought conditions were monitored using the Standardized Precipitation-Evapotranspiration Index calculated at short-term using one month accumulation period for is calculation (SPEI-1). SPEI-1 time series for each Brazilian urban city were obtained based on the Climatology and Climate Laboratory (LCSC) datasets.
- Daily mean temperature information was obtained from the fifth European Centre for Medium-range Weather Forecasts (ECMWF) reanalysis — ERA5. Datasets were processed to obtain weekly time series.
- The authors like to thank the Unified Health System of Brazil, the Climatology and Climate Laboratory and the European Centre for Medium-range Weather Forecasts reanalysis to allow using the health and climate information required to conduct this study.
- This study was financially supported by the ED481B-2021-122 grant from the Xunta de Galicia, Spain. I was partially supported by the projects from the Xunta of Galicia, Spain (ED431C 2021/44), FCT [([PTDC/CTA − CLI/28902/2017](https://www.sciencedirect.com/science/article/pii/S2212094721000189#gs1)) and [UIDB/50019/2020](https://www.sciencedirect.com/science/article/pii/S2212094721000189#gs1) - Instituto Dom Luiz], and grants from CNPq (305159/2018–6), FAPERJ (E26/202.714/2019), and PIBIC-UFRJ (grant 148598/2020-0)
- The data used in this study are available through Salvador, C., Vicedo-Cabrera, A.M., Libonati, R., Russo, A., Garcia, B.N., Belem, L.B.C., Gimeno, L., Nieto, R (2022). Effects of Drought on Mortality in Macro Urban Areas of Brazil between 2000 to 2019: Dataset [Dataset]. Mendeley Data, V1. <http://dx.doi.org/10.17632/bbj27mxx9g.1>
- The authors declare no conflict of interest relevant to this study

**Table of Contents**

**Supplementary methods**

**Method S1.** Quasi-Poisson equation to estimate the short-term effects of drought on non-external, circulatory, and respiratory causes of weekly mortality in each Brazilian location between 2000 to 2019.

**Supplementary Tables and Figures**

**Figure S1**. Weekly temporal evolution of the Standardized Precipitation-Evapotranspiration Index calculated at one month of accumulation (SPEI-1) in the Development Integrated Region of the Federal District and Surrounding Areas (RIDE-DF) between 2000 and 2019.

**Table S1**. Summary of the total quasi-likelihood Akaike information Criteria considering different modelling approaches to control the SPE-1, mean temperature, and seasonal and long-term trend.

**Table S2.** Comparison of the overall association between drought separated by categories of severity and non-external mortality (relative risks (RRs) and attributable fractions (AF (%) with their respective 95% confidence intervals) using the main model and the following best-fit model according to the quasi-Akaike criteria information after changing the control of seasonal and long-term trend.

**Figure S2:** Additional analysis. Comparison of an overall association between drought and non-external mortality expressed as relative risk and 95% confidence intervals per 1-unit increase in drought conditions using threshold=-0.84 (M1, qAIC =180165.3), and threshold= 0 (M2, qAIC =180196) to establish drought onset, as well as using a nonlinear function (M3, qAIC c=180397). The same control of the seasonal and long-term trend was applied in the different approaches.

**Table S3.** P-values obtained in the Chi-square test to assess the interaction between the effect of drought events and temperature on non-external cause mortality for each Brazilian location.

**Figure S3**. Scatter plot of the weekly non-external mortality and weekly SPEI-1 series in each Brazilian location of study from 2000 to 2019.

**Table S4.** Descriptive analysis corresponding to weekly counts of no-external, circulatory, and respiratory mortality causes of the total population, males, and females separated by age ranges (all ages, 0-9, 10-44, 45-64, 65-74, ≥75 years old) in each Brazilian location and overall, between 2000 to 2019.

**Table S5.** Number of drought events (in weeks) measured by the Standardized Precipitation-Evapotranspiration Index obtained at short-term (SPEI-1) per year of study period for each Brazilian location between 2000 to 2019.

**Table S6.** Relative risks and 95% confidence intervals of weekly non-external mortality of the population separated by sex and age ranges associated with moderate drought in each Brazilian location.

**Table S7.** Relative risks and 95% confidence intervals of weekly non-external mortality of the population separated by sex and age ranges associated with severe drought in each Brazilian location.

**Table S8.** Relative risks and 95% confidence intervals of weekly non-external mortality of the population separated by sex and age ranges associated with extreme drought in each Brazilian location.

**Table S9.** Relative risks and 95% confidence intervals of weekly specific cause mortality of the population separated by sex and age ranges associated with moderate (M), severe (S), extreme (E) drought in each Brazilian location.

**Figure S4.** The overall association between drought and non-external mortality expressed as relative risks (RRs) (red) and 95% confidence intervals (grey area) when the lag dimension was extended to 3 weeks in the different population groups.

**Method S1.** Quasi-Poisson equation to estimate the short-term effects of drought on non-external, circulatory, and respiratory causes of weekly mortality in each Brazilian location between 2000 to 2019.


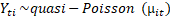


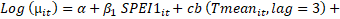


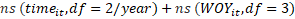


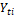
 corresponds to the number of mortality outcomes on week *t* in location *i* (independently for each cause of death and age group in the total, male and female populations);
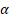
 is the intercept;
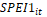
refers to the continuous series of drought index obtained at one month of accumulation, where we applied a threshold parametrization (threshold=-0.84) during the same week of exposure; *cb* is the crossbasis function of mean temperature (Tmean) during 0-3 lag weeks, including three internal knots at the 10^th^, 75^th^, 90^th^ percentiles of each mean temperature series. The adjustment of the seasonal and long-trend is also represented by a natural spline function of time with 2 degrees of freedom per year of study and another natural spline function of an indicator of the week of the year (WOY) with three degrees of freedom.


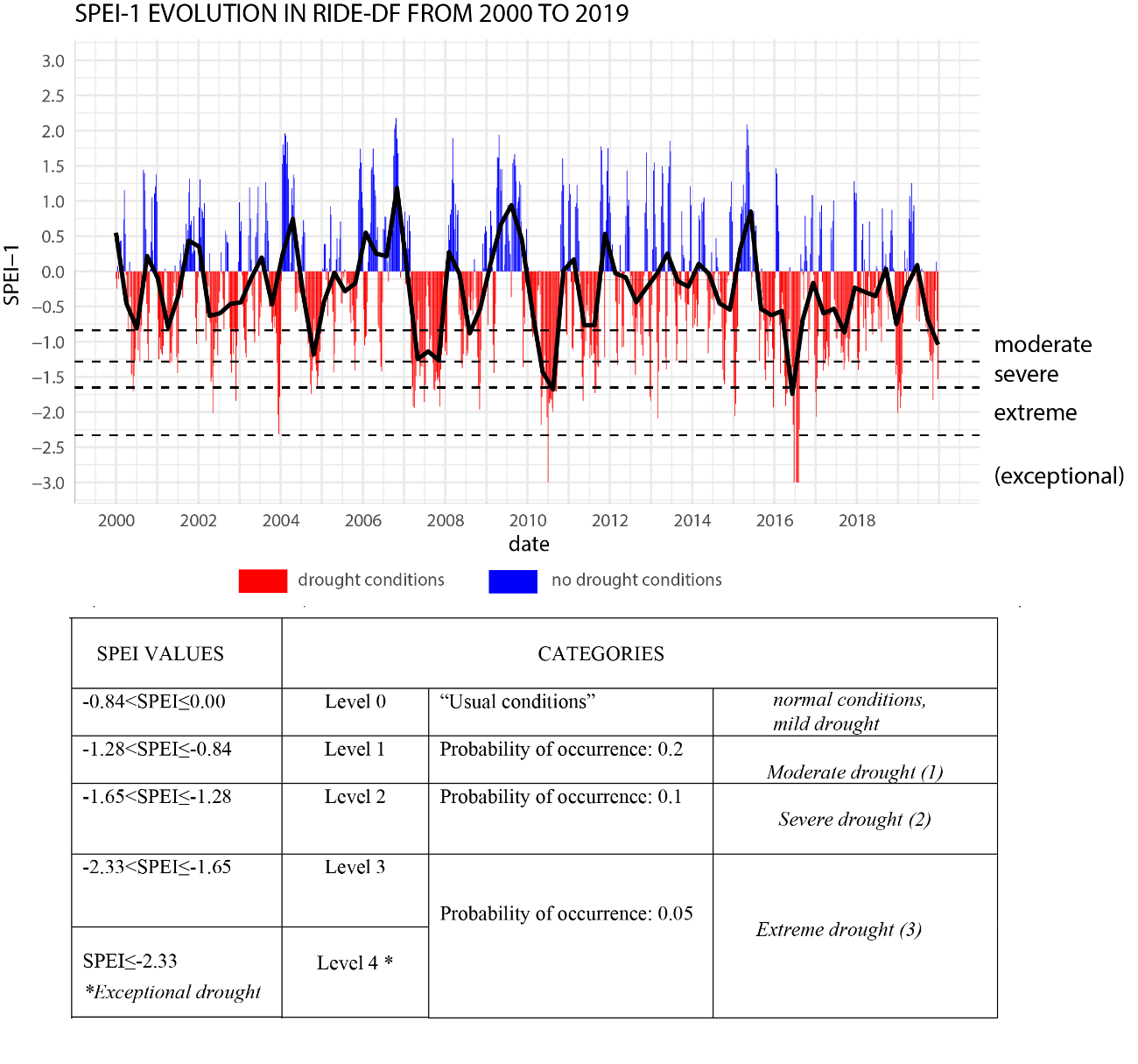


**Figure S1.** Weekly temporal evolution of the Standardized Precipitation-Evapotranspiration Index calculated at one month of accumulation (SPEI-1) in the Development Integrated Region of the Federal District and Surrounding Areas (RIDE-DF) between 2000 and 2019. The different thresholds that allow the classification of drought severity in moderate, severe, and extreme are also represented, following the criteria of Agnew (2000) and the Climatology and Climate Services Laboratory (https://lcsc.csic.es).

**Table S1**. Summary of the total quasi-likelihood Akaike information Criteria considering different modelling approaches to control the SPE1, mean temperature, and seasonal and long-term trend.

| **SPEI1, cbTmean,**  **Time component**  **(Without week of the year component)** | | **SPEI1** | | | | **Time component** |
| --- | --- | --- | --- | --- | --- | --- |
|  |  | **Threshold= 0** | | **Threshold= -0.84** | |  |
| Tmean | Exp-resp: ns //percentiles (10^th^, 50^th^, 90^th^ )  Lag-response: integer (lag3) | 184843.2 | | 184809 | | df=4*year |
|  | Exp-resp: bs //percentiles (10^th^, 50^th^, 90^th^ )  Lag-response: integer (lag3) | 185165.5 | | 185133 | |  |
|  | Exp-resp: ns //percentiles (10^th^, 50^th^, 90^th^ )  Lag-response: strata, breaks=1 (lag3) | 185497.1 | | 185444.2 | |  |
|  | Exp-resp: bs //percentiles (10^th^, 50^th^, 90^th^ )  Lag-response: strata, breaks=1 (lag3) | 185630.5 | | 185575 | |  |
|  | Exp-resp: ns //percentiles (10^th^, 50^th^, 90^th^ )  Lag-response: integer (lag3) | 186472.8 | | 186448.9 | | df=5*year |
|  | Exp-resp: bs //percentiles (10^th^, 50^th^, 90^th^ )  Lag-response: integer (lag3) | 186803.2 | | 186782.6 | |  |
|  | Exp-resp: ns //percentiles (10^th^, 50^th^, 90^th^ )  Lag-response: strata, breaks=1 (lag3) | 187045.6 | | 187007.1 | |  |
|  | Exp-resp: bs //percentiles (10^th^, 50^th^, 90^th^ )  Lag-response: strata, breaks=1 (lag3) | 187190.4 | | 187151.1 | |  |
|  | Exp-resp: ns //percentiles (10^th^, 50^th^, 90^th^ )  Lag-response: integer (lag3) | 188358.7 | | 188340.7 | | df=6*year |
|  | Exp-resp: bs //percentiles (10^th^, 50^th^, 90^th^ )  Lag-response: integer (lag3) | 188680.4 | | 188667.7 | |  |
|  | Exp-resp: ns //percentiles (10^th^, 50^th^, 90^th^ )  Lag-response: strata, breaks=1 (lag3) | 188906.1 | | 1888771 | |  |
|  | Exp-resp: bs //percentiles (10^th^, 50^th^, 90^th^ )  Lag-response: strata, breaks=1 (lag3) | 189048.9 | | 189019.8 | |  |
|  | Exp-resp: ns //percentiles (10^th^, 50^th^, 90^th^ )  Lag-response: integer (lag3) | 190966.4 | | 190952 | | df=7*year |
|  | Exp-resp: bs //percentiles (10^th^, 50^th^, 90^th^ )  Lag-response: integer (lag3) | 191250.3 | | 191247.2 | |  |
|  | Exp-resp: ns //percentiles (10^th^, 50^th^, 90^th^ )  Lag-response: strata, breaks=1 (lag3) | 191629.4 | | 191596 | |  |
|  | Exp-resp: bs //percentiles (10^th^, 50^th^, 90^th^ )  Lag-response: strata, breaks=1 (lag3) | 191736.6 | | 191708.5 | |  |
|  | Exp-resp: ns //percentiles (10^th^, 75^th^,90^th^ )  Lag-response: integer (lag3) | 184801 | | 184773 | | df=4*year |
|  | Exp-resp: bs //percentiles (10^th^, 75^th^,90^th^ )  Lag-response: integer (lag3) | 185084.2 | | 185053 | |  |
|  | Exp-resp: ns //percentiles (10^th^, 75^th^,90^th^ )  Lag-response: strata, breaks=1 (lag3) | 185413.1 | | 185363.3 | |  |
|  | Exp-resp: bs //percentiles (10^th^, 75^th^,90^th^ )  Lag-response: strata, breaks=1 (lag3) | 185574.7 | | 185519.1 | |  |
|  | Exp-resp: ns //percentiles (10^th^, 75^th^,90^th^ )  Lag-response: integer (lag3) | 168413.6 | | 1863941 | | df=5*year |
|  | Exp-resp: bs //percentiles (10^th^, 75^th^,90^th^ )  Lag-response: integer (lag3) | 186721.8 | | 186700.3 | |  |
|  | Exp-resp: ns //percentiles (10^th^, 75^th^,90^th^ )  Lag-response: strata, breaks=1 (lag3) | 186956.1 | | 186918.2 | |  |
|  | Exp-resp: bs //percentiles (10^th^, 75^th^,90^th^ )  Lag-response: strata, breaks=1 (lag3) | 187129.4 | | 187088.4 | |  |
|  | Exp-resp: ns //percentiles (10^th^, 75^th^,90^th^ )  Lag-response: integer (lag3) | 188248.5 | | 188235.2 | | df=6*year |
|  | Exp-resp: bs //percentiles (10^th^, 75^th^,90^th^ )  Lag-response: integer (lag3) | 188629.2 | | 188616.9 | |  |
|  | Exp-resp: ns //percentiles (10^th^, 75^th^,90^th^ )  Lag-response: strata, breaks=1 (lag3) | 188777.3 | | 188749 | |  |
|  | Exp-resp: bs //percentiles (10^th^, 75^th^,90^th^ )  Lag-response: strata, breaks=1 (lag3) | 189006.1 | | 188975.6 | |  |
|  | Exp-resp: ns //percentiles (10^th^, 75^th^,90^th^ )  Lag-response: integer (lag3) | 190873.6 | | 190864.8 | | df=7*year |
|  | Exp-resp: bs //percentiles (10^th^, 75^th^,90^th^ )  Lag-response: integer (lag3) | 191235.3 | | 191230.2 | |  |
|  | Exp-resp: ns //percentiles (10^th^, 75^th^,90^th^ )  Lag-response: strata, breaks=1 (lag3) | 191516.3 | | 191483.6 | |  |
|  | Exp-resp: bs //percentiles (10^th^, 75^th^,90^th^ )  Lag-response: strata, breaks=1 (lag3) | 191728.2 | | 191696.2 | |  |
| **SPEI1, cbTmean,**  **Time component**  **week of the year component (as lineal term)** | | **SPEI1** | | | | **Time component** |
|  |  | **Threshold= 0** | | **Threshold= -0.84** | |  |
| Tmean | Exp-resp: ns //percentiles (10^th^, 50^th^, 90^th^ )  Lag-response: integer (lag3) | 183025 | | 182964.4 | | df=2*year |
|  | Exp-resp: bs //percentiles (10^th^, 50^th^, 90^th^ )  Lag-response: integer (lag3) | 183351.7 | | 183294.7 | |  |
|  | Exp-resp: ns //percentiles (10^th^, 50^th^, 90^th^ )  Lag-response: strata, breaks=1 (lag3) | 183855 | | 183790.8 | |  |
|  | Exp-resp: bs //percentiles (10^th^, 50^th^, 90^th^ )  Lag-response: strata, breaks=1 (lag3) | 183926 | | 183867.1 | |  |
|  | Exp-resp: ns //percentiles (10^th^, 50^th^, 90^th^ )  Lag-response: integer (lag3) | 183459.6 | | 183401.4 | | df=3*year |
|  | Exp-resp: bs //percentiles (10^th^, 50^th^, 90^th^ )  Lag-response: integer (lag3) | 183823.2 | | 183769.3 | |  |
|  | Exp-resp: ns //percentiles (10^th^, 50^th^, 90^th^ )  Lag-response: strata, breaks=1 (lag3) | 184189.9 | | 184110.3 | |  |
|  | Exp-resp: bs //percentiles (10^th^, 50^th^, 90^th^ )  Lag-response: strata, breaks=1 (lag3) | 184461.1 | | 184410.7 | |  |
|  | Exp-resp: ns //percentiles (10^th^, 50^th^, 90^th^ )  Lag-response: integer (lag3) | 184739.4 | | 184702.6 | | df=4*year |
|  | Exp-resp: bs //percentiles (10^th^, 50^th^, 90^th^ )  Lag-response: integer (lag3) | 185079.9 | | 185044.1 | |  |
|  | Exp-resp: ns //percentiles (10^th^, 50^th^, 90^th^ )  Lag-response: strata, breaks=1 (lag3) | 185408.7 | | 185352.6 | |  |
|  | Exp-resp: bs //percentiles (10^th^, 50^th^, 90^th^ )  Lag-response: strata, breaks=1 (lag3) | 185803.2 | | 185763.7 | |  |
|  | Exp-resp: ns //percentiles (10^th^, 75^th^, 90^th^ )  Lag-response: integer (lag3) | 183026.6 | | 182972.2 | | df=2*year |
|  | Exp-resp: bs //percentiles (10^th^, 75^th^, 90^th^ )  Lag-response: integer (lag3) | 183357.5 | | 183302.6 | |  |
|  | Exp-resp: ns//percentiles (10^th^, 75^th^, 90^th^ )  Lag-response: strata, breaks=1 (lag3) | 183805.1 | | 183744.6 | |  |
|  | Exp-resp: bs //percentiles (10^th^, 75^th^, 90^th^ )  Lag-response: strata, breaks=1 (lag3) | 183994.4 | | 183934.5 | |  |
|  | Exp-resp: ns //percentiles (10^th^, 75^th^, 90^th)^ Lag-response: integer (lag3) | 183448.7 | | 183397.3 | | df=3*year |
|  | Exp-resp: bs //percentiles (10^th^, 75^th^, 90^th^ )  Lag-response: integer (lag3) | 183788.8 | | 183737 | |  |
|  | Exp-resp: ns //percentiles (10^th^, 75^th^, 90^th^ )  Lag-response: strata, breaks=1 (lag3) | 184126.6 | | 184050 | |  |
|  | Exp-resp: bs //percentiles (10^th^, 75^th^, 90^th)^ Lag-response: strata, breaks=1 (lag3) | 184445 | | 184445 | |  |
|  | Exp-resp: ns //percentiles (10^th^, 75^th^,90^th^ )  Lag-response: integer (lag3) | 184709.8 | | 184678.4 | | df=4*year |
|  | Exp-resp: bs //percentiles (10^th^, 75^th^,90^th^ )  Lag-response: integer (lag3) | 184999.6 | | 184965.5 | |  |
|  | Exp-resp: ns //percentiles (10^th^, 75^th^,90^th^ )  Lag-response: strata, breaks=1 (lag3) | 185335 | | 185281.6 | |  |
|  | Exp-resp: bs //percentiles (10^th^, 75^th^,90^th^ )  Lag-response: strata, breaks=1 (lag3) | 185787.1 | | 185747.5 | |  |
| **SPEI1, cbTmean,**  **Time component**  **week of the year component (ns function, df=2)** | | **SPEI1** | | | | **Time component** |
|  |  | **Threshold= 0** | | **Threshold= -0.84** | |  |
| Tmean | Exp-resp: ns //percentiles (10^th^, 50^th^, 90^th^ )  Lag-response: integer (lag3) | 180988.7 | | 180966.9 | | df=2*year |
|  | Exp-resp: bs //percentiles (10^th^, 50^th^, 90^th^ )  Lag-response: integer (lag3) | 181273 | | 181250.3 | |  |
|  | Exp-resp: ns //percentiles (10^th^, 50^th^, 90^th^ )  Lag-response: strata, breaks=1 (lag3) | 181530.7 | | 181510.2 | |  |
|  | Exp-resp: bs //percentiles (10^th^, 50^th^, 90^th^ )  Lag-response: strata, breaks=1 (lag3) | 181646.3 | | 181620.4 | |  |
|  | Exp-resp: ns //percentiles (10^th^, 50^th^, 90^th^ )  Lag-response: integer (lag3) | 180885.6 | | 180864.1 | | df=3*year |
|  | Exp-resp: bs //percentiles (10^th^, 50^th^, 90^th^ )  Lag-response: integer (lag3) | 181128.2 | | 181107.2 | |  |
|  | Exp-resp: ns //percentiles (10^th^, 50^th^, 90^th^ )  Lag-response: strata, breaks=1 (lag3) | 181432.2 | | 181401.6 | |  |
|  | Exp-resp: bs //percentiles (10^th^, 50^th^, 90^th^ )  Lag-response: strata, breaks=1 (lag3) | 181523.9 | | 181488.8 | |  |
|  | Exp-resp: ns //percentiles (10^th^, 50^th^, 90^th^ )  Lag-response: integer (lag3) | 182549.6 | | 182539.9 | | df=4*year |
|  | Exp-resp: bs //percentiles (10^th^, 50^th^, 90^th^ )  Lag-response: integer (lag3) | 182802.8 | | 182791.1 | |  |
|  | Exp-resp: ns //percentiles (10^th^, 50^th^, 90^th^ )  Lag-response: strata, breaks=1 (lag3) | 183158 | | 183133.8 | |  |
|  | Exp-resp: bs //percentiles (10^th^, 50^th^, 90^th^ )  Lag-response: strata, breaks=1 (lag3) | 183260.4 | | 183230.2 | |  |
|  | Exp-resp: ns //percentiles (10^th^, 75^th^, 90^th^ )  Lag-response: integer (lag3) | 180926.5 | | 180909.4 | | df=2*year |
|  | Exp-resp: bs // percentiles (10^th^, 75^th^, 90^th^ )  Lag-response: integer (lag3) | 181235.2 | | 181215.3 | |  |
|  | Exp-resp: ns // percentiles (10^th^, 75^th^, 90^th^ )  Lag-response: strata, breaks=1 (lag3) | 181445.6 | | 181426.9 | |  |
|  | Exp-resp: bs // percentiles (10^th^, 75^th^, 90^th^ )  Lag-response: strata, breaks=1 (lag3) | 181623.9 | | 181600.3 | |  |
|  | Exp-resp: ns // percentiles (10^th^, 75^th^, 90^th^ )  Lag-response: integer (lag3) | 180782 | | 180767.2 | | df=3*year |
|  | Exp-resp: bs // percentiles (10^th^, 75^th^, 90^th^ )  Lag-response: integer (lag3) | 181057.1 | | 181037.3 | |  |
|  | Exp-resp: ns // percentiles (10^th^, 75^th^, 90^th^ )  Lag-response: strata, breaks=1 (lag3) | 181323.9 | | 181295.9 | |  |
|  | Exp-resp: bs // percentiles (10^th^, 75^th^, 90^th^ )  Lag-response: strata, breaks=1 (lag3) | 181467.8 | | 181433.3 | |  |
|  | Exp-resp: ns // percentiles (10^th^, 75^th^, 90^th^ )  Lag-response: integer (lag3) | 182438.3 | | 182432.3 | | df=4*year |
|  | Exp-resp: bs // percentiles (10^th^, 75^th^, 90^th^ )  Lag-response: integer (lag3) | 182736.5 | | 182727 | |  |
|  | Exp-resp: ns // percentiles (10^th^, 75^th^, 90^th^ )  Lag-response: strata, breaks=1 (lag3) | 183037.4 | | 183014.1 | |  |
|  | Exp-resp: bs // percentiles (10^th^, 75^th^, 90^th^ )  Lag-response: strata, breaks=1 (lag3) | 183208.5 | | 183179.3 | |  |
| **SPEI1, cbTmean,**  **Time component**  **Week of the year component (ns function, df=3)** | | **SPEI1** | | | | **Time component** |
|  |  | **Threshold= 0** | | **Threshold= -0.84** | |  |
| Tmean | Exp-resp: ns //percentiles (10^th^, 50^th^, 90^th^ )  Lag-response: integer (lag3) | 180257.9 | | 180222.2 | | df=2*year |
|  | Exp-resp: bs //percentiles (10^th^, 50^th^, 90^th^ )  Lag-response: integer (lag3) | 180553.6 | | 180517.2 | |  |
|  | Exp-resp: ns //percentiles (10^th^, 50^th^, 90^th^ )  Lag-response: strata, breaks=1 (lag3) | 180744.6 | | 180701.4 | |  |
|  | Exp-resp: bs //percentiles (10^th^, 50^th^, 90^th^ )  Lag-response: strata, breaks=1 (lag3) | 180864.2 | | 180815.4 | |  |
|  | Exp-resp: ns //percentiles (10^th^, 50^th^, 90^th^ )  Lag-response: integer (lag3) | 181084.8 | | 181061.5 | | df=3*year |
|  | Exp-resp: bs //percentiles (10^th^, 50^th^, 90^th^ )  Lag-response: integer (lag3) | 181338.3 | | 181315.5 | |  |
|  | Exp-resp: ns //percentiles (10^th^, 50^th^, 90^th^ )  Lag-response: strata, breaks=1 (lag3) | 181626.1 | | 181593.8 | |  |
|  | Exp-resp: bs //percentiles (10^th^, 50^th^, 90^th^ )  Lag-response: strata, breaks=1 (lag3) | 181728.9 | | 181691.8 | |  |
|  | Exp-resp: ns //percentiles (10^th^, 50^th^, 90^th^ )  Lag-response: integer (lag3) | 182565.2 | | 182559.3 | | df=4*year |
|  | Exp-resp: bs //percentiles (10^th^, 50^th^, 90^th^ )  Lag-response: integer (lag3) | 182811.1 | | 182804.8 | |  |
|  | Exp-resp: ns //percentiles (10^th^, 50^th^, 90^th^ )  Lag-response: strata, breaks=1 (lag3) | 183130.2 | | 183110.7 | |  |
|  | Exp-resp: bs //percentiles (10^th^, 50^th^, 90^th^ )  Lag-response: strata, breaks=1 (lag3) | 183219.6 | | 183195.9 | |  |
|  | Exp-resp: ns // percentiles (10^th^, 75^th^, 90^th^ )  Lag-response: integer (lag3) | 180196.3 | | **180165.3** | | df=2*year |
|  | Exp-resp: bs // percentiles (10^th^, 75^th^, 90^th^ )  Lag-response: integer (lag3) | 180517.2 | | 180440.1 | |  |
|  | Exp-resp: ns // percentiles (10^th^, 75^th^, 90^th^ )  Lag-response: strata, breaks=1 (lag3) | 180659.2 | | 180617.8 | |  |
|  | Exp-resp: bs // percentiles (10^th^, 75^th^, 90^th^ )  Lag-response: strata, breaks=1 (lag3) | 180806.3 | | 180760.2 | |  |
|  | Exp-resp: ns // percentiles (10^th^, 75^th^, 90^th^ )  Lag-response: integer (lag3) | 180986.3 | | 180969.6 | | df=3*year |
|  | Exp-resp: bs // percentiles (10^th^, 75^th^, 90^th^ )  Lag-response: integer (lag3) | 181315.5 | | 181239.7 | |  |
|  | Exp-resp: ns // percentiles (10^th^, 75^th^, 90^th^ )  Lag-response: strata, breaks=1 (lag3) | 181522.1 | | 181492.5 | |  |
|  | Exp-resp: bs // percentiles (10^th^, 75^th^, 90^th^ )  Lag-response: strata, breaks=1 (lag3) | 181666.9 | | 181630.5 | |  |
|  | Exp-resp: ns // percentiles (10^th^, 75^th^, 90^th^ )  Lag-response: integer (lag3) | 182465.3 | | 182463.1 | | df=4*year |
|  | Exp-resp: bs //percentiles (10^th^, 75^th^,90^th^ )  Lag-response: integer (lag3) | 182804.8 | | 182752.7 | |  |
|  | Exp-resp: ns //percentiles (10^th^, 75^th^,90^th^ )  Lag-response: strata, breaks=1 (lag3) | 183015.6 | | 182997 | |  |
|  | Exp-resp: bs //percentiles (10^th^, 75^th^,90^th^ )  Lag-response: strata, breaks=1 (lag3) | 183180.1 | | 183155.9 | |  |
| **SPEI1, cbTmean,**  **Time component** | | **SPEI1** | | | | **Time component** |
|  |  | **ns,**  **knots= 10th, 50th percentiles** | **bs,**  **knots= 10th, 50th percentiles** | **ns,**  **knots= 10th, 50^th^, 90th percentile** | **bs,**  **knots= 10th, 50^th^, 90th percentiles** |  |
| Tmean | Exp-resp: ns //percentiles (10^th^, 50^th^, 90^th^ )  Lag-response: integer (lag3) | 185043.9 | 185112.7 | 185094.6 | 185244.3 | df=4*year |
|  | Exp-resp: bs //percentiles (10^th^, 50^th^, 90^th^ )  Lag-response: integer (lag3) | 185381.9 | 185429.4 | 185423.7 | 185560.6 |  |
|  | Exp-resp: ns //percentiles (10^th^, 50^th^, 90^th^ )  Lag-response: strata, breaks=1 (lag3) | 185671.4 | 185745.7 | 185719.8 | 185885.8 |  |
|  | Exp-resp: bs //percentiles (10^th^, 50^th^, 90^th^ )  Lag-response: strata, breaks=1 (lag3) | 185814.1 | 185875.1 | 185857.8 | 186015.1 |  |
|  | Exp-resp: ns //percentiles (10^th^, 50^th^, 90^th^ )  Lag-response: integer (lag3) | 186721.6 | 186787.4 | 186773.9 | 186920.5 | df=5*year |
|  | Exp-resp: bs //percentiles (10^th^, 50^th^, 90^th^ )  Lag-response: integer (lag3) | 187063 | 187110.2 | 187100.4 | 187243.1 |  |
|  | Exp-resp: ns //percentiles (10^th^, 50^th^, 90^th^ )  Lag-response: strata, breaks=1 (lag3) | 187272.8 | 187342.4 | 187320.9 | 187484.8 |  |
|  | Exp-resp: bs //percentiles (10^th^, 50^th^, 90^th^ )  Lag-response: strata, breaks=1 (lag3) | 187422.7 | 187480.4 | 187462.3 | 187621.1 |  |
|  | Exp-resp: ns //percentiles (10^th^, 50^th^, 90^th^ )  Lag-response: integer (lag3) | 188612.4 | 188675.6 | 188646.8 | 188803.9 | df=6*year |
|  | Exp-resp: bs //percentiles (10^th^, 50^th^, 90^th^ )  Lag-response: integer (lag3) | 188948 | 188990.3 | 188963.3 | 189118.6 |  |
|  | Exp-resp: ns //percentiles (10^th^, 50^th^, 90^th^ )  Lag-response: strata, breaks=1 (lag3) | 189148.5 | 189213.8 | 189176.1 | 189353.1 |  |
|  | Exp-resp: bs //percentiles (10^th^, 50^th^, 90^th^ )  Lag-response: strata, breaks=1 (lag3) | 189296 | 189347.4 | 189312.9 | 192140.4 |  |
|  | Exp-resp: ns // percentiles (10^th^, 50^th^, 90^th^ )  Lag-response: integer (lag3) | 191222.8 | 191251.3 | 191224.9 | 191365.9 | df=7*year |
|  | Exp-resp: bs // percentiles (10^th^, 50^th^, 90^th^ )  Lag-response: integer (lag3) | 191524.9 | 191538.6 | 191508.6 | 191650.1 |  |
|  | Exp-resp: ns // percentiles (10^th^, 50^th^, 90^th^ )  Lag-response: strata, breaks=1 (lag3) | 191875.7 | 191911.4 | 191872.1 | 192038.6 |  |
|  | Exp-resp: bs // percentiles (10^th^, 50^th^, 90^th^ )  Lag-response: strata, breaks=1 (lag3) | 191990.6 | 192017.7 | 191978.2 | 192140.4 |  |
|  | Exp-resp: ns // percentiles (10^th^, 75^th^, 90^th^ )  Lag-response: integer (lag3) | 185013.6 | 185070.7 | 185063.1 | 185206.6 | df=4*year |
|  | Exp-resp: bs // percentiles (10^th^, 75^th^, 90^th^ )  Lag-response: integer (lag3) | 185306.6 | 185349.9 | 185346.7 | 185480 |  |
|  | Exp-resp: ns // percentiles (10^th^, 75^th^, 90^th^ )  Lag-response: strata, breaks=1 (lag3) | 185595 | 185664.2 | 185647.8 | 185807 |  |
|  | Exp-resp: bs // percentiles (10^th^, 75^th^, 90^th^ )  Lag-response: strata, breaks=1 (lag3) | 185760.8 | 185823.6 | 185805.5 | 185964.2 |  |
|  | Exp-resp: ns // percentiles (10^th^, 75^th^, 90^th^ )  Lag-response: integer (lag3) | 186667.7 | 186724.7 | 186719.6 | 186861.6 | df=5*year |
|  | Exp-resp: bs // percentiles (10^th^, 75^th^, 90^th^ )  Lag-response: integer (lag3) | 186984.9 | 187029 | 187022.6 | 187162.5 |  |
|  | Exp-resp: ns // percentiles (10^th^, 75^th^, 90^th^ )  Lag-response: strata, breaks=1 (lag3) | 187185.6 | 187252.9 | 187240.2 | 187398.8 |  |
|  | Exp-resp: bs // percentiles (10^th^, 75^th^, 90^th^ )  Lag-response: strata, breaks=1 (lag3) | 187362.9 | 187423.5 | 187405.5 | 187565.3 |  |
|  | Exp-resp: ns // percentiles (10^th^, 75^th^, 90^th^ )  Lag-response: integer (lag3) | 188514.1 | 188567.8 | 188547.4 | 188699.6 | df=6*year |
|  | Exp-resp: bs // percentiles (10^th^, 75^th^, 90^th^ )  Lag-response: integer (lag3) | 188897.6 | 188938.3 | 188915.7 | 189068 |  |
|  | Exp-resp: ns // percentiles (10^th^, 75^th^, 90^th^ )  Lag-response: strata, breaks=1 (lag3) | 189027.4 | 189090.5 | 189061.9 | 189233.4 |  |
|  | Exp-resp: bs // percentiles (10^th^, 75^th^, 90^th^ )  Lag-response: strata, breaks=1 (lag3) | 189252.1 | 189306.9 | 189273.8 | 189446.3 |  |
|  | Exp-resp: ns // percentiles (10^th^, 75^th^, 90^th^ )  Lag-response: integer (lag3) | 191140.3 | 191159.7 | 191141.3 | 191279.4 | df=7*year |
|  | Exp-resp: bs //percentiles (10^th^, 75^th^, 90^th^ )  Lag-response: integer (lag3) | 191508.8 | 191516.7 | 191492.6 | 191630 |  |
|  | Exp-resp: ns //percentiles (10^th^, 75^th^,90^th^ )  Lag-response: strata, breaks=1 (lag3) | 191769 | 191804.3 | 191773.4 | 191936.1 |  |
|  | Exp-resp: bs //percentiles (10^th^, 75^th^,90^th^ )  Lag-response: strata, breaks=1 (lag3 | 191977 | 192004.2 | 191967.6 | 192129.5 |  |
| **SPEI1, cbTmean,**  **Time component**  **week of the year component (as lineal term)** | | **SPEI1** | | | | **Time component** |
|  |  | **ns**  **knots= 10th, 50th percentiles** | **bs**  **knots= 10th, 50th percentiles** | **ns,**  **knots= 10th, 50^th^, 90th percentiles** | **bs,**  **knots= 10th, 50^th^, 90th percentiles** |  |
| Tmean | Exp-resp: ns //percentiles (10^th^, 50^th^, 90^th^ )  Lag-response: integer (lag3) | 184945.1 | 1850073 | 184994.3 | 185134.2 | df=4*year |
|  | Exp-resp: bs //percentiles (10^th^, 50^th^, 90^th^ )  Lag-response: integer (lag3) | 185298.4 | 185337.6 | 185339.3 | 185463.6 |  |
|  | Exp-resp: ns //percentiles (10^th^, 50^th^, 90^th^ )  Lag-response: strata, breaks=1 (lag3) | 185587.2 | 185658.7 | 185634 | 185794.1 |  |
|  | Exp-resp: bs //percentiles (10^th^, 50^th^, 90^th^ )  Lag-response: strata, breaks=1 (lag3) | 185736.3 | 185794.3 | 185779.3 | 185930 |  |
|  | Exp-resp: ns //percentiles (10^th^, 50^th^, 90^th^ )  Lag-response: integer (lag3) | 186746.7 | 186807.1 | 186800.4 | 186940.3 | df=5*year |
|  | Exp-resp: bs //percentiles (10^th^, 50^th^, 90^th^ )  Lag-response: integer (lag3) | 187085.2 | 187124.8 | 187124.7 | 187257.9 |  |
|  | Exp-resp: ns //percentiles (10^th^, 50^th^, 90^th^ )  Lag-response: strata, breaks=1 (lag3) | 187327.7 | 187394.9 | 187377.5 | 187537.4 |  |
|  | Exp-resp: bs //percentiles (10^th^, 50^th^, 90^th^ )  Lag-response: strata, breaks=1 (lag3) | 187471.2 | 187526.2 | 187513 | 187667.9 |  |
|  | Exp-resp: ns //percentiles (10^th^, 50^th^, 90^th^ )  Lag-response: integer (lag3) | 188652.2 | 188709.4 | 188686.5 | 188839.8 | df=6*year |
|  | Exp-resp: bs //percentiles (10^th^, 50^th^, 90^th^ )  Lag-response: integer (lag3) | 188980.2 | 189013.1 | 188995.3 | 189144.2 |  |
|  | Exp-resp: ns //percentiles (10^th^, 50^th^, 90^th^ )  Lag-response: strata, breaks=1 (lag3) | 189217.9 | 189279.7 | 189245.6 | 189421.6 |  |
|  | Exp-resp: bs //percentiles (10^th^, 50^th^, 90^th^ )  Lag-response: strata, breaks=1 (lag3) | 189355.8 | 189402.7 | 189372.8 | 189543.7 |  |
|  | Exp-resp: ns // percentiles (10^th^, 50^th^, 90^th^ )  Lag-response: integer (lag3) | 191285.3 | 191306.4 | 191287.8 | 191425.1 | df=7*year |
|  | Exp-resp: bs // percentiles (10^th^, 50^th^, 90^th^ )  Lag-response: integer (lag3) | 191580.3 | 191583.2 | 191564.5 | 191700.2 |  |
|  | Exp-resp: ns // percentiles (10^th^, 50^th^, 90^th^ )  Lag-response: strata, breaks=1 (lag3) | 191961.2 | 191992.5 | 191957.7 | 192123.7 |  |
|  | Exp-resp: bs // percentiles (10^th^, 50^th^, 90^th^ )  Lag-response: strata, breaks=1 (lag3) | 192069.8 | 192092 | 192057.8 | 192220.7 |  |
|  | Exp-resp: ns // percentiles (10^th^, 75^th^, 90^th^ )  Lag-response: integer (lag3) | 184925.8 | 184975.6 | 184974.4 | 185105.8 | df=4*year |
|  | Exp-resp: bs // percentiles (10^th^, 75^th^, 90^th^ )  Lag-response: integer (lag3) | 188222.3 | 185257.6 | 185263 | 185381.6 |  |
|  | Exp-resp: ns // percentiles (10^th^, 75^th^, 90^th^ )  Lag-response: strata, breaks=1 (lag3) | 185520.1 | 185586.1 | 185572 | 185724.2 |  |
|  | Exp-resp: bs // percentiles (10^th^, 75^th^, 90^th^ )  Lag-response: strata, breaks=1 (lag3) | 185679.7 | 185739.3 | 185723.5 | 185874.8 |  |
|  | Exp-resp: ns // percentiles (10^th^, 75^th^, 90^th^ )  Lag-response: integer (lag3) | 186696.4 | 186747.4 | 186750 | 186884.5 | df=5*year |
|  | Exp-resp: bs // percentiles (10^th^, 75^th^, 90^th^ )  Lag-response: integer (lag3) | 187019.3 | 187056 | 187059.7 | 187188.3 |  |
|  | Exp-resp: ns // percentiles (10^th^, 75^th^, 90^th^ )  Lag-response: strata, breaks=1 (lag3) | 187242.9 | 187307.6 | 187299.4 | 187453.8 |  |
|  | Exp-resp: bs // percentiles (10^th^, 75^th^, 90^th^ )  Lag-response: strata, breaks=1 (lag3) | 187419.1 | 187477.3 | 187464.1 | 187619.1 |  |
|  | Exp-resp: ns // percentiles (10^th^, 75^th^, 90^th^ )  Lag-response: integer (lag3) | 188555.3 | 188602.2 | 188588.6 | 188736.8 | df=6*year |
|  | Exp-resp: bs // percentiles (10^th^, 75^th^, 90^th^ )  Lag-response: integer (lag3) | 188944 | 188975.6 | 188962 | 189106.6 |  |
|  | Exp-resp: ns // percentiles (10^th^, 75^th^, 90^th^ )  Lag-response: strata, breaks=1 (lag3) | 189097.6 | 189157.1 | 189132.4 | 189303 |  |
|  | Exp-resp: bs // percentiles (10^th^, 75^th^, 90^th^ )  Lag-response: strata, breaks=1 (lag3) | 189320.8 | 189371.6 | 189343.1 | 189513.7 |  |
|  | Exp-resp: ns // percentiles (10^th^, 75^th^, 90^th^ )  Lag-response: integer (lag3) | 191203.8 | 191214.6 | 191204.9 | 191338.7 | df=7*year |
|  | Exp-resp: bs //percentiles (10^th^, 75^th^, 90^th^ )  Lag-response: integer (lag3) | 191564.8 | 191561.5 | 191549.6 | 191679.7 |  |
|  | Exp-resp: ns //percentiles (10^th^, 75^th^,90^th^ )  Lag-response: strata, breaks=1 (lag3) | 191855.6 | 191886.3 | 191860.3 | 192022.3 |  |
|  | Exp-resp: bs //percentiles (10^th^, 75^th^,90^th^ )  Lag-response: strata, breaks=1 (lag3) | 192054 | 192076.4 | 192945.2 | 192206.7 |  |
| **SPEI1, cbTmean,**  **Time component**  **Week of the year component (ns, df=2)** | | **SPEI1** | | | | **Time component** |
|  |  | **ns,**  **knots= 10th, 50th percentiles** | **bs,**  **knots= 10th, 50th percentiles** | **ns,**  **knots= 10th, 50^th^, 90th percentiles** | **bs,**  **knots= 10th, 50^th^, 90th percentiles** |  |
| Tmean | Exp-resp: ns //percentiles (10^th^, 50^th^, 90^th^ )  Lag-response: integer (lag3) | 181205.4 | 181263.1 | 181257.8 | 181383.2 | df=2*year |
|  | Exp-resp: bs //percentiles (10^th^, 50^th^, 90^th^ )  Lag-response: integer (lag3) | 181501 | 181544.7 | 181550 | 181671.8 |  |
|  | Exp-resp: ns //percentiles (10^th^, 50^th^, 90^th^ )  Lag-response: strata, breaks=1 (lag3) | 181724.9 | 181792.8 | 181780 | 181921.2 |  |
|  | Exp-resp: bs //percentiles (10^th^, 50^th^, 90^th^ )  Lag-response: strata, breaks=1 (lag3) | 181844.7 | 181903.5 | 181899.8 | 182039.6 |  |
|  | Exp-resp: ns //percentiles (10^th^, 50^th^, 90^th^ )  Lag-response: integer (lag3) | 181112 | 184160.3 | 181157.9 | 181291.1 | df=3*year |
|  | Exp-resp: bs //percentiles (10^th^, 50^th^, 90^th^ )  Lag-response: integer (lag3) | 181363 | 181389.6 | 181400.1 | 181522.3 |  |
|  | Exp-resp: ns //percentiles (10^th^, 50^th^, 90^th^ )  Lag-response: strata, breaks=1 (lag3) | 181644.8 | 181703.5 | 181689.8 | 181842.3 |  |
|  | Exp-resp: bs //percentiles (10^th^, 50^th^, 90^th^ )  Lag-response: strata, breaks=1 (lag3) | 181739.3 | 181785.8 | 181781 | 181927.9 |  |
|  | Exp-resp: ns //percentiles (10^th^, 50^th^, 90^th^ )  Lag-response: integer (lag3) | 182788.7 | 182834.8 | 182837.7 | 182975.4 | df=4*year |
|  | Exp-resp: bs //percentiles (10^th^, 50^th^, 90^th^ )  Lag-response: integer (lag3) | 183048.8 | 183066.1 | 183087.6 | 183205.4 |  |
|  | Exp-resp: ns //percentiles (10^th^, 50^th^, 90^th^ )  Lag-response: strata, breaks=1 (lag3) | 183381.6 | 183440.8 | 183428.4 | 183588.9 |  |
|  | Exp-resp: bs //percentiles (10^th^, 50^th^, 90^th^ )  Lag-response: strata, breaks=1 (lag3) | 183484.6 | 183526.9 | 183527 | 183675.7 |  |
|  | Exp-resp: ns // percentiles (10^th^, 75^th^, 90^th^ )  Lag-response: integer (lag3) | 181150 | 181197.7 | 181203.3 | 181321.9 | df=2*year |
|  | Exp-resp: bs // percentiles (10^th^, 75^th^, 90^th^ )  Lag-response: integer (lag3) | 181462.2 | 181506 | 181509.9 | 181628.4 |  |
|  | Exp-resp: ns // percentiles (10^th^, 75^th^, 90^th^ )  Lag-response: strata, breaks=1 (lag3) | 181644 | 181707.4 | 181704.6 | 18838.5 |  |
|  | Exp-resp: bs // percentiles (10^th^, 75^th^, 90^th^ )  Lag-response: strata, breaks=1 (lag3) | 181820 | 181880.7 | 181875.1 | 182013.5 |  |
|  | Exp-resp: ns // percentiles (10^th^, 75^th^, 90^th^ )  Lag-response: integer (lag3) | 181016.6 | 181051.3 | 181062.3 | 181184.9 | df=3*year |
|  | Exp-resp: bs // percentiles (10^th^, 75^th^, 90^th^ )  Lag-response: integer (lag3) | 181292.4 | 181320.4 | 181330.1 | 181450.3 |  |
|  | Exp-resp: ns // percentiles (10^th^, 75^th^, 90^th^ )  Lag-response: strata, breaks=1 (lag3) | 181539.8 | 181592.1 | 181590.4 | 181732 |  |
|  | Exp-resp: bs // percentiles (10^th^, 75^th^, 90^th^ )  Lag-response: strata, breaks=1 (lag3) | 181680.3 | 181730.6 | 181722.8 | 181870.3 |  |
|  | Exp-resp: ns // percentiles (10^th^, 75^th^, 90^th^ )  Lag-response: integer (lag3) | 182687.5 | 182718.8 | 182734.7 | 182861.6 | df=4*year |
|  | Exp-resp: bs // percentiles (10^th^, 75^th^, 90^th^ )  Lag-response: integer (lag3) | 182983.3 | 182999.4 | 183022.4 | 183137.8 |  |
|  | Exp-resp: ns // percentiles (10^th^, 75^th^, 90^th^ )  Lag-response: strata, breaks=1 (lag3) | 183266.6 | 183318.7 | 183318.1 | 183467.8 |  |
|  | Exp-resp: bs // percentiles (10^th^, 75^th^, 90^th^ )  Lag-response: strata, breaks=1 (lag3) | 183431.6 | 183478.5 | 183475.3 | 183626.5 |  |
| **SPEI1, cbTmean,**  **Time component**  **Week of the year component (ns, df=3)** | | **SPEI1** | | | | **Time component** |
|  |  | **ns,**  **knots= 10th, 50th percentiles** | **bs,**  **knots= 10th, 50th percentiles** | **ns,**  **knots= 10th, 50^th^, 90th percentiles** | **bs,**  **knots= 10th, 50^th^, 90th percentiles** |  |
| Tmean | Exp-resp: ns //percentiles (10^th^, 50^th^, 90^th^ )  Lag-response: integer (lag3) | 180453 | 180494.3 | 180492.9 | 180613.1 | df=2*year |
|  | Exp-resp: bs //percentiles (10^th^, 50^th^, 90^th^ )  Lag-response: integer (lag3) | 180761.9 | 180787.4 | 180797.3 | 180909.8 |  |
|  | Exp-resp: ns //percentiles (10^th^, 50^th^, 90^th^ )  Lag-response: strata, breaks=1 (lag3) | 180918.5 | 180971.8 | 180960.7 | 181101.1 |  |
|  | Exp-resp: bs //percentiles (10^th^, 50^th^, 90^th^ )  Lag-response: strata, breaks=1 (lag3) | 181044 | 181088.4 | 181085.3 | 181223 |  |
|  | Exp-resp: ns //percentiles (10^th^, 50^th^, 90^th^ )  Lag-response: integer (lag3) | 181310.3 | 181361.3 | 181357.3 | 181491.5 | df=3*year |
|  | Exp-resp: bs //percentiles (10^th^, 50^th^, 90^th^ )  Lag-response: integer (lag3) | 181572.5 | 181602.1 | 181612 | 181735.3 |  |
|  | Exp-resp: ns //percentiles (10^th^, 50^th^, 90^th^ )  Lag-response: strata, breaks=1 (lag3) | 181835.7 | 181896.9 | 181881.6 | 182035.3 |  |
|  | Exp-resp: bs //percentiles (10^th^, 50^th^, 90^th^ )  Lag-response: strata, breaks=1 (lag3) | 181941.4 | 181990.4 | 181985.2 | 182133 |  |
|  | Exp-resp: ns //percentiles (10^th^, 50^th^, 90^th^ )  Lag-response: integer (lag3) | 182812.1 | 183858.1 | 182854.1 | 182994.4 | df=4*year |
|  | Exp-resp: bs //percentiles (10^th^, 50^th^, 90^th^ )  Lag-response: integer (lag3) | 183064.4 | 183081 | 183093.3 | 183216.3 |  |
|  | Exp-resp: ns //percentiles (10^th^, 50^th^, 90^th^ )  Lag-response: strata, breaks=1 (lag3) | 183359.5 | 183417.7 | 183398 | 183561.4 |  |
|  | Exp-resp: bs //percentiles (10^th^, 50^th^, 90^th^ )  Lag-response: strata, breaks=1 (lag3) | 183449.5 | 183490.5 | 183481.2 | 183635 |  |
|  | Exp-resp: ns // percentiles (10^th^, 75^th^, 90^th^ )  Lag-response: integer (lag3) | 180397 | 180426.5 | 180437.3 | 180548.8 | df=2*year |
|  | Exp-resp: bs // percentiles (10^th^, 75^th^, 90^th^ )  Lag-response: integer (lag3) | 180679.8 | 180705.8 | 180715 | 180825.4 |  |
|  | Exp-resp: ns // percentiles (10^th^, 75^th^, 90^th^ )  Lag-response: strata, breaks=1 (lag3) | 180835 | 180883.1 | 180883.1 | 181014.7 |  |
|  | Exp-resp: bs // percentiles (10^th^, 75^th^, 90^th^ )  Lag-response: strata, breaks=1 (lag3) | 180982.3 | 181029.8 | 181024.7 | 181162.7 |  |
|  | Exp-resp: ns // percentiles (10^th^, 75^th^, 90^th^ )  Lag-response: integer (lag3) | 181219.6 | 181256.9 | 181266.9 | 181390.4 | df=3*year |
|  | Exp-resp: bs // percentiles (10^th^, 75^th^, 90^th^ )  Lag-response: integer (lag3) | 181495.9 | 181527.2 | 181535.8 | 181657.9 |  |
|  | Exp-resp: ns // percentiles (10^th^, 75^th^, 90^th^ )  Lag-response: strata, breaks=1 (lag3) | 181735 | 181789.6 | 181787 | 181929.7 |  |
|  | Exp-resp: bs // percentiles (10^th^, 75^th^, 90^th^ )  Lag-response: strata, breaks=1 (lag3) | 181876.2 | 181929 | 181920.5 | 182069.3 |  |
|  | Exp-resp: ns // percentiles (10^th^, 75^th^, 90^th^ )  Lag-response: integer (lag3) | 182720.9 | 182753.2 | 182762.3 | 182892.5 | df=4*year |
|  | Exp-resp: bs // percentiles (10^th^, 75^th^, 90^th^ )  Lag-response: integer (lag3) | 183011.6 | 183026.5 | 183041.4 | 183161.1 |  |
|  | Exp-resp: ns // percentiles (10^th^, 75^th^, 90^th^ )  Lag-response: strata, breaks=1 (lag3) | 183249.9 | 183302.2 | 183294.4 | 183448 |  |
|  | Exp-resp: bs // percentiles (10^th^, 75^th^, 90^th^ )  Lag-response: strata, breaks=1 (lag3) | 183408.4 | 183453.2 | 183441.8 | 183597 |  |

**Table S2.** Comparison of the overall association between drought separated by categories of severity and non-external mortality (relative risks (RRs) and attributable fractions (AF (%) with their respective 95% confidence intervals) using the main model and the following best-fit model according to the quasi-Akaike criteria information after changing the control of seasonal and long-term trend. RR: Relative Risk; AF: Attributable Fraction

| **NON-EXTERNAL CAUSES OF MORTALITY** | | | | | | |
| --- | --- | --- | --- | --- | --- | --- |
| **Population groups** | **M1: moderate drought** | **M1: severe drought** | **M1: extreme drought** | **M2: moderate drought** | **M2: severe drought** | **M2: extreme drought** |
| **Total (all ages)** | RR:1.003 [0.999, 1.007]  AF: 0.30 [-0.10, 0.70] | RR: 1.006 [0.998, 1.013]  AF: 0.60 [-0.20, 1.28] | RR: 1.010 [0.996, 1.025]  FA: 0.99 [-0.40, 2.44] | RR:1.004 [1.000, 1.008]  AF: 0.40 [0, 0.79] | RR:1.007 [0.999, 1.015]  AF: 0.70 [-0.10, 1.48] | RR:1.014 [0.999, 1.029]  AF: 1.38 [-0.10, 2.82] |
| **Total (0—9)** | RR:1.008 [0.999, 1.017]  AF: 0.79 (-0.10, 1.67) | RR: 1.015 [0.999, 1.032]  AF:1.48 [-0.1, 3.10] | RR: 1.029 [0.998, 1.061]  FA: 2.82 [-0.20, 5.75] | RR:1.010 [1.001, 1.018]  AF: 0.99 [0.10, 1.77] | RR:1.018 [1.001, 1.035]  AF:1.77[0.10, 3.38] | RR:1.033 [1.002, 1.065]  AF:3.19 [0.20, 6.10] |
| **Total (10—44)** | RR:0.998 [0.991, 1.005]  AF: -0.20 [-0.91, 0.50] | RR: 0.996 [0.984, 1.009]  AF: -0.40 [-1.63, 0.89] | RR: 0.993 [0.971, 1.016]  FA: -0.70 [-2.99, 1.57] | RR:0.998 [0.992, 1.005]  AF: -0.20 [-0.81, 0.50] | RR:0.997 [0.985, 1.01]  AF: -0.30 [-1.52, 0.99] | RR:0.995 [0.972, 1.018]  AF: -0.50 [-2.88, 1.77] |
| **Total (45—64)** | RR: 1.001 [0.996, 1.006]  AF: 0.10 [-0.40, 0.60] | RR: 1.002 [0.992, 1.011]  AF: 0.20 [-0.81, 1.09] | RR: 1.003 [0.986, 1.021]  FA: 0.3 [-1.42, 2.06] | RR:1.002 [0.997, 1.007]  AF: 0.20 [-0.30, 0.70] | RR:1.004 [0.994, 1.014]  AF:0.40 [-0.60, 1.38] | RR:1.007 [0.989, 1.025]  AF:0.70 [-1.11, 2.44] |
| **Total (65—74)** | RR:1.005 [0.999, 1.01]  AF: 0.50 [-0.10, 0.99] | RR:1.009 [0.999, 1.019]  AF: 0.89 [-0.10, 1.86] | RR: 1.016 [0.997, 1.035]  FA: 1.57 [-0.30,3.38] | RR:1.005 [0.999, 1.011]  AF:0.50 [-0.10, 1.09] | RR:1.010 [0.999, 1.021]  AF:0.99 [-0.10, 2.06] | RR:1.018 [0.998, 1.038]  AF:1.77 [-0.20, 3.66] |
| **Total ( ≥75)** | RR:1.005 [1.000, 1.010]  AF:0.50 [0, 0.99] | RR:1.009 [1.000, 1.018]  AF:0.89 [0, 1.77] | RR: 1.016 [0.999, 1.033]  FA: 1.57 [-0.10, 3.19] | RR:1.006 [1.001, 1.011]  AF:0.60 [0.10, 1.09] | RR:1.011 [1.001, 1.020]  AF:1.09 [0.10, 1.96] | RR:1.020 [1.002, 1.038]  AF:1.96 [0.20, 3.66] |
| **Males (all ages)** | RR:1.001 [0.997, 1.006]  AF:0.10 [-0.30, 0.60] | RR:1.003 [0.994, 1.011]  AF:0.30 [-0.60, 1.09] | RR: 1.005 [0.989, 1.021]  FA: 0.50 [-1.11, 2.06] | RR:1.002 [0.997, 1.007]  AF:0.20 [-0.30, 0.70] | RR:1.003 [0.995, 1.012]  AF:0.30 [-0.50, 1.19] | RR:1.006 [0.990, 1.023]  AF:0.60 [-1.01, 2.25] |
| **Males (0—9)** | RR:1.009 [0.998, 1.019]  AF: 0.89 [-0.20, 1.86] | RR:1.016 [0.996, 1.036]  AF:1.57 [-0.40, 3.47] | RR: 1.030[0.993, 1.068]  FA: 2.91 [-0.70, 6.37] | RR:1.010 [0.998, 1.021]  AF:0.99 [-0.20, 2.06] | RR:1.018 [0.997, 1.029]  AF:1.77 [-0.30, 2.82] | RR:1.033 [0.995, 1.073]  AF:3.19 [-0.50, 6.80] |
| **Males (10—44)** | RR:0.995 [0.987, 1.003]  AF: -0.50 [-1.32, 0.30] | RR:0.991 [0.977, 1.005]  AF: -0.91 [-2.35, 0.50] | RR: 0.983 [0.957, 1.010]  FA: -1.73 [-4.49, 0.99] | RR:0.997 [0.989, 1.005]  AF: -0.30 [-1.11, 0.50] | RR: 0.995 [0.980, 1.011]  AF: -0.50 [-2.04, 1.09] | RR:0.990 [0.963, 1.018]  AF: -1.01 [-3.84, 1.77] |
| **Males 45—64** | RR:1.000 [0.994, 1.005]  AF: 0 [-0.60, 0.50] | RR:0.999 [0.989, 1.010]  AF: -0.10 [-1.11, 0.99] | RR: 0.999 [0.979, 1.019]  FA: -0.1 [-2.15, 1.86] | RR:1.000 [0.994, 1.006]  AF:0 [-0.60, 0.60] | RR:0.999 [0.988, 1.011]  AF: -0.10 [-1.21, 1.09] | RR:0.999 [0.979, 1.02]  AF: -0.10 [-2.14, 1.96] |
| **Males 65—74** | RR:1.001 [0.995, 1.007]  AF: 0.10 [-0.50, 0.70] | RR:1.001 [0.990, 1.013]  AF:0.10 [-1.01, 1.28] | RR: 1.003 [0.981, 1.025]  FA: 0.3 [-1.94, 2.44] | RR:1.001 [0.995, 1.008]  AF:0.10 [-0.50, 0.79] | RR:1.002 [0.990, 1.015]  AF:0.20 [-0.10, 2.15] | RR:1.004 [0.982, 1.027]  AF:0.40 [-1.83, 2.63] |
| **Males ≥75** | RR:1.005 [0.999, 1.011]  AF: 0.50 [-0.10, 1.09] | RR:1.010 [0.999, 1.021]  AF:0.99 [-1.01, 2.06] | RR: 1.018 [0.998, 1.039]  FA: 1.77 [-0.20, 3.75] | RR:1.005 [0.999, 1.012]  AF:0.50 [-0.10, 1.19] | RR:1.01 [0.999, 1.022]  AF:0.99 [-0.10, 2.15] | RR:1.019 [0.998, 1.040]  AF:1.86 [-0.20, 3.85] |
| **Females (all)** | RR:1.005 [1.000, 1.009]  AF: 0.50 [0, 0.89] | RR:1.009 [1.000, 1.018]  AF:0.89 [0, 1.77] | RR:1.017 [1.000, 1.033]  FA: 1.67 [0, 3.19] | RR:1.006 [1.001, 1.011]  AF:0.60 [0.10, 1.09] | RR:1.011 [1.002, 1.020]  AF:1.09 [0.20, 1.96] | RR:1.021 [1.004, 1.038]  AF:2.06 [0.40, 3.66] |
| **Females 0—9** | RR:1.008 [0.996, 1.021]  AF: 0.79 [-0.40, 2.06] | RR:1.016 [0.992, 1.039]  AF:1.57 [-0.81, 3.75] | RR: 1.029 [0.986, 1.074]  FA: 2.68 [-1.42, 6.89] | RR:1.010 [0.998, 1.022]  AF:0.99 [-0.20, 2.15] | RR:1.019 [0.997, 1.041]  AF:1.86 [-0.30, 3.94] | RR:1.035[0.994, 1.078]  AF:3.38 [-0.60, 7.24] |
| **Females 10—44** | RR:1.003 [0.994, 1.012]  AF:0.30 [-0.60, 1.19] | RR:1.005 [0.989, 1.022]  AF:0.50 [-1.11, 2.15] | RR: 1.010 [0.979, 1.041]  FA: 0.99 [-2.15, 3.94] | RR:1.001 [0.992, 1.010]  AF:0.10 [-0.81, 0.99] | RR:1.002 [0.985, 1.020]  AF:0.20 [-1.52, 1.96] | RR:1.004 [0.972, 1.036]  AF:0.40 [-2.88, 3.47] |
| **Females 45—64** | RR:1.004 [0998, 1.010]  AF: 0.40 [-0.20, 0.99] | RR:1.007 [0.995, 1.019]  AF:0.70 [-0.50, 1.86] | RR: 1.014 [0.992, 1.036]  FA: 1.38 [-0.81, 3.47] | RR:1.006 [0.999, 1.013]  AF:0.60 [-0.10, 1.28] | RR:1.011 [0.999, 1.024]  AF:1.09 [-0.10, 2.34] | RR:1.021 [0.998, 1.044]  AF:2.06 [-0.20, 4.21] |
| **Females 65—64** | RR:1.009 [1.003, 1.016]  AF: 0.89 [0.30, 1.57] | RR:1.018 [1.005, 1.03]  AF: 1.77 [0.50, 2.91] | RR: 1.033 [1.009, 1.057]  FA: 3.19 [0.89, 5.39] | RR:1.010 [1.003, 1.017]  AF:0.99 [0.30, 1.67] | RR:1.018 [1.005, 1.032]  AF: 1.77 [0.50, 3.10] | RR:1.034 [1.010, 1.059]  AF:3.29 [0.99, 5.57] |
| **Females ≥75** | RR:1.004 [0.999, 1.010]  AF: 0.40 [-0.10, 0.99] | RR:1.008 [0.998, 1.019]  AF:0.79 [-0.20, 1.86] | RR: 1.015 [0.996, 1.035]  FA: 1.48 [-0.40, 3.38] | RR:1.006 [1.000, 1.012]  AF:0.60 [0, 1.19] | RR:1.011 [1.001, 1.022]  AF:1.09 [0.10, 2.15] | RR:1.021 [1.001, 1.041]  AF:2.06 [0.10, 3.94] |
| **CIRCULATORY CAUSES OF MORTALITY** | | | | | | |
| **Population groups** | **M1: moderate drought** | **M1: severe drought** | **M1: extreme drought** | **M2: moderate drought** | **M2: severe drought** | **M2: extreme drought** |
| **Total (all ages)** | RR: 1.002 [0.997, 1.007]  AF: 0.20 [-0.30, 0.70] | RR:1.005 [0.995, 1.014]  AF:0.50 [-0.50, 1.38] | RR: 1.008 [0.991, 1.026]  AF: 0.79 [-0.91, 2.53] | RR:1.004 [0.999, 1.009]  AF: 0.40 [-0.10, 0.89] | RR:1.007 [0.998, 1.017]  AF: 0.70 [-0.20, 1.67] | RR:1.013 [0.995, 1.032]  AF:1.28 [-0.50, 3.100] |
| **Males (all ages)** | RR:0.998[0.989, 1.007]  AF: -0.20 [-1.11, 0.70] | RR:0.996 [0.979, 1.013]  AF: -0.40 [-2.15, 1.28] | RR: 0.993 [0.961, 1.025]  AF: -0.70 [-4.06, 2.44] | RR:1.000 [0.993, 1.007]  AF:0 [-0.70, 0.70] | RR:1.000 [0.987, 1.014]  AF:0 [-1.32, 1.38] | RR:1.000 [0.976, 1.025]  AF:0 [-2.46, 2.44] |
| **Females (all ages)** | RR:1.007 [1.001, 1.013]  AF: 0.70 [0.10, 1.28] | RR:1.013 [1.002, 1.024]  AF: 1.28 [0.20, 2.34] | RR: 1.025 [1.004, 1.046]  AF:2.44 [0.40, 4.40] | RR:1.008 [1.002, 1.014]  AF:0.79 [0.200, 1.38] | RR:1.015 [1.004, 1.027]  AF:1.48 [0.40, 2.63] | RR:1.029 [1.007, 1.051]  AF:2.82 [0.70, 4.85] |
| **RESPIRATORY CAUSES OF MORTALITY** | | | | | | |
| **Population groups** | **M1: moderate** | **M1: severe** | **M1: extreme** | **M2: moderate** | **M2: severe** | **M2: extreme** |
| **Total (all ages)** | RR: 1.004 [0.995, 1.013]  AF: 0.40 [-0.50, 1.28] | RR:1.007 [0.991, 1.023]  AF:0.70 [-0.91, 2.25] | RR: 1.013 [0.983, 1.044]  AF: 1.28 [-1.73, 4.21] | RR:1.007 [0.999, 1.015]  AF:0.70 [-0.10, 1.48] | RR:1.013 [0.997, 1.028]  AF:1.28 [-0.30, 2.72] | RR:1.024 [0.995, 1.053]  AF: 2.34 [-0.50, 5.03] |
| **Males (all ages)** | RR:0.999 [0.989, 1.009]  AF: -0.10 [-1.11, 0.89] | RR:0.998 [0.979, 1.017]  AF: -0.20 [-2.15, 1.67] | RR: 0.996 [0.962, 1.031]  AF: -0.40 [-3.95, 3.01] | RR:1.001 [0.991, 1.011]  AF:0.100 [0.50, 2.15] | RR:1.003 [0.984, 1.021]  AF:0.30 [-1.63, 2.06] | RR:1.005 [0.971, 1.040]  AF:0.50 [-2.99, 3.85] |
| **Females (all ages)** | RR:1.011 [1.002, 1.020]  AF:1.09 [0.20, 1.96] | RR:1.020 [1.004, 1.037]  AF: 1.96 [0.40, 3.57] | RR: 1.038 [1.007, 1.070]  AF:3.66 [0.70, 6.54] | RR:1.014 [1.005, 1.022]  AF:1.38 [0.50, 2.15] | RR:1.026 [1.010, 1.042]  AF:2.53 [0.99, 4.03] | RR:1.048 [1.018, 1.079]  AF:4.58 [1.77, 7.32] |

**
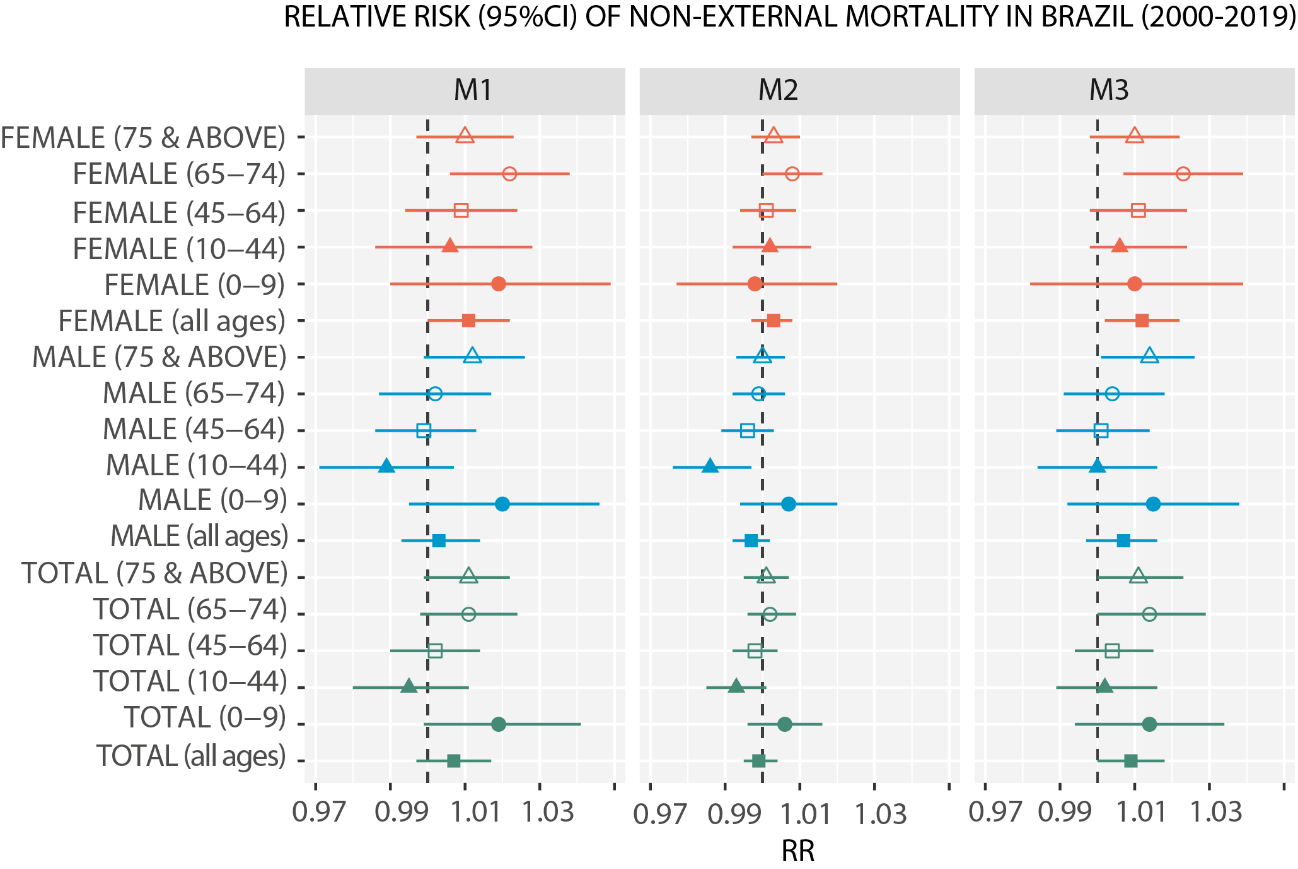
**

**Figure S2:** Additional analysis. Comparison of an overall association between drought and non-external mortality expressed as relative risk and 95% confidence intervals per 1-unit increase in drought conditions using threshold=-0.84 (M1, qAIC =180165.3), and threshold= 0 (M2, qAIC =180196) to establish drought onset, as well as using a nonlinear function (M3, qAIC c=180397). The same control of the seasonal and long-term trend was applied in the different approaches.

**Table S3.** P-values obtained in the Chi-square test to assess the interaction between the effect of drought events and temperature on non-external cause mortality for each Brazilian location.

| **REGION** | **P-VALOR (CHI-SQUARE TEST)** |
| --- | --- |
| Manaus | 0.49 |
| Belem | 0.09 |
| Fortaleza | 0.72 |
| Recife | 0.44 |
| Salvador | 0.38 |
| RIDE-DF | 0.65 |
| Cuiaba | 0.70 |
| Campo Grande | 0.25 |
| Rio de Janeiro | 0.56 |
| São Paulo | 0.77 |
| Curitiba | 0.70 |
| Florianopolis | 0.40 |
| Porto Alegre | 0.44 |


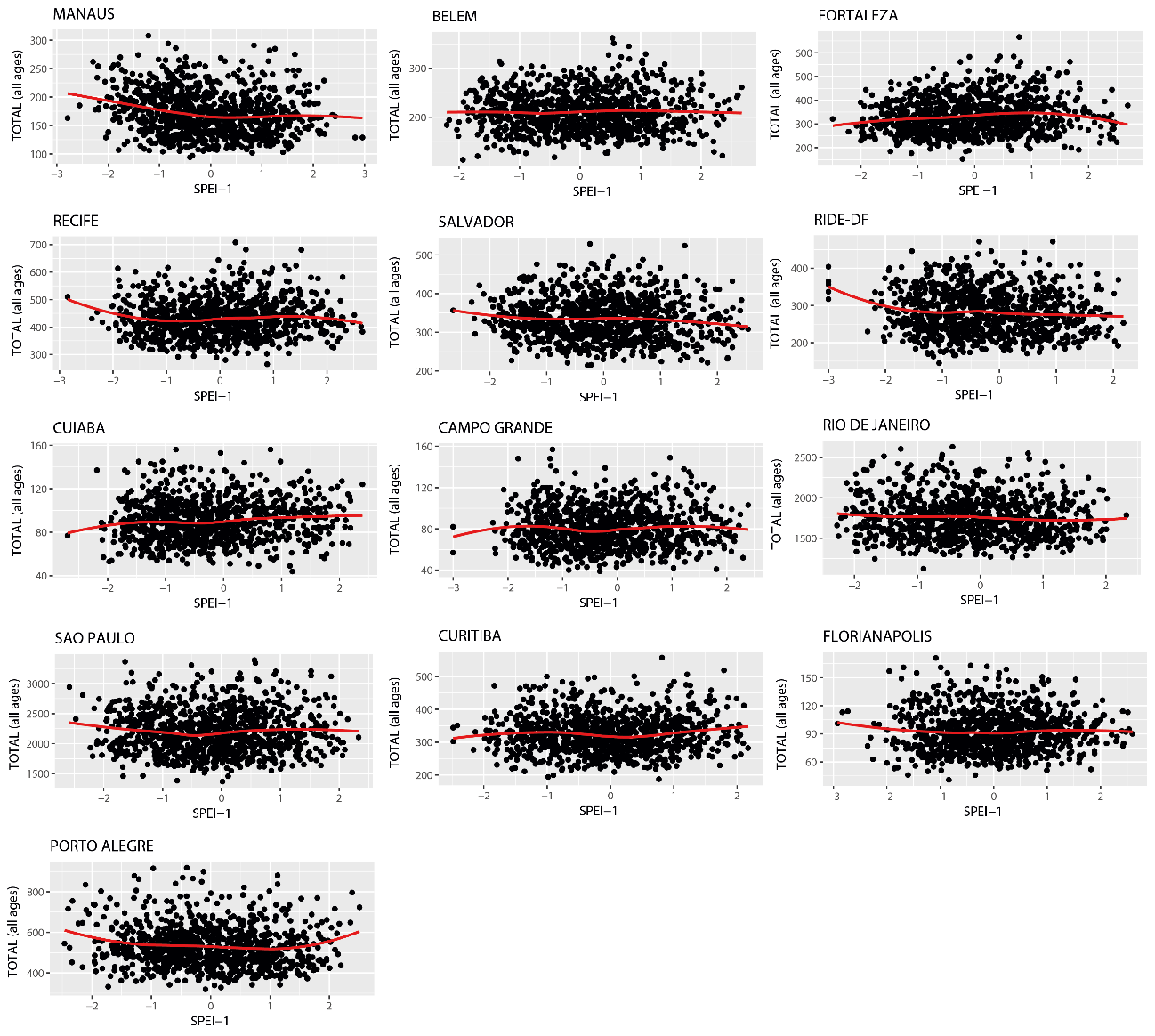


**Figure S3.** Scatter plot of the weekly non-external mortality and weekly SPEI-1 series in each Brazilian location of study from 2000 to 2019.

**Table S4­.** Descriptive analysis corresponding to weekly counts of no-external, circulatory, and respiratory mortality causes of the total population, males, and females separated by age ranges (all ages, 0-9, 10-44, 45-64, 65-74, ≥75 years old) in each Brazilian location and overall, between 2000 to 2019.

| AGE  GROUPS | REGION | GROUP | MORTALITY CAUSES | | |
| --- | --- | --- | --- | --- | --- |
|  |  |  | **NATURAL** | **CIRCULATORY** | **RESPIRATORY** |
| All ages | MR MANAUS | Total | 164123 | 33985 | 15740 |
|  |  | Males | 89325 | 18561 | 8213 |
|  |  | Females | 74798 | 15424 | 7527 |
|  | MR BELEM | Total | 202455 | 55291 | 29590 |
|  |  | Males | 104989 | 28911 | 14679 |
|  |  | Females | 97466 | 26380 | 14911 |
|  | MR FORTALEZA | Total | 319439 | 90565 | 39645 |
|  |  | Males | 163433 | 46550 | 18900 |
|  |  | Females | 156006 | 44015 | 20745 |
|  | MR RECIFE | Total | 413415 | 144760 | 57472 |
|  |  | Males | 207262 | 72443 | 27171 |
|  |  | Females | 206153 | 72317 | 30301 |
|  | MR SALVADOR | Total | 320557 | 100405 | 37851 |
|  |  | Males | 161346 | 51214 | 18747 |
|  |  | Females | 159211 | 49191 | 19104 |
|  | RIDE-DF | Total | 269597 | 85760 | 27377 |
|  |  | Males | 145933 | 46554 | 14119 |
|  |  | Females | 123664 | 39206 | 13258 |
|  | MR CUIABA | Total | 86770 | 27353 | 10145 |
|  |  | Males | 48807 | 15359 | 5683 |
|  |  | Females | 37963 | 11994 | 4462 |
|  | CAMPO GRANDE | Total | 77312 | 26939 | 16605 |
|  |  | Males | 42105 | 14958 | 11505 |
|  |  | Females | 35207 | 11981 | 5100 |
|  | MR RIO DE JANEIRO | Total | 1682979 | 539703 | 214108 |
|  |  | Males | 840089 | 269633 | 105778 |
|  |  | Females | 842890 | 270070 | 108330 |
|  | MR SÃO PAULO | Total | 2111312 | 763774 | 293136 |
|  |  | Males | 1101180 | 394760 | 153030 |
|  |  | Females | 1010132 | 369014 | 140106 |
|  | MR CURITIBA | Total | 312112 | 102516 | 37306 |
|  |  | Males | 163810 | 53125 | 18399 |
|  |  | Females | 148302 | 49391 | 18907 |
|  | MR FLORIANOPOLIS | Total | 88393 | 31204 | 10409 |
|  |  | Males | 47612 | 16682 | 5595 |
|  |  | Females | 40781 | 14522 | 4814 |
|  | MR PORTO ALEGRE | Total | 510375 | 158446 | 66909 |
|  |  | Males | 260632 | 76874 | 34643 |
|  |  | Females | 249743 | 81572 | 32266 |
| TOTAL | | **Total** | **6558839** | **2160701** | **856293** |
|  |  | **Males** | **3376523** | **1105624** | **436462** |
|  |  | **Females** | **3182316** | **1055077** | **419831** |
| 0-9 | MR MANAUS | Total | 18741 | 199 | 1520 |
|  |  | Males | 10344 | 97 | 846 |
|  |  | Females | 8397 | 102 | 674 |
|  | MR BELEM | Total | 16458 | 217 | 1475 |
|  |  | Males | 9101 | 105 | 754 |
|  |  | Females | 7357 | 112 | 721 |
|  | MR FORTALEZA | Total | 19452 | 401 | 1492 |
|  |  | Males | 10760 | 218 | 831 |
|  |  | Females | 8692 | 183 | 661 |
|  | MR RECIFE | Total | 19098 | 215 | 1528 |
|  |  | Males | 10562 | 118 | 846 |
|  |  | Females | 8536 | 97 | 682 |
|  | MR SALVADOR | Total | 22503 | 862 | 1749 |
|  |  | Males | 12265 | 673 | 925 |
|  |  | Females | 10238 | 189 | 824 |
|  | RIDE-DF | Total | 19129 | 260 | 1030 |
|  |  | Males | 10537 | 137 | 560 |
|  |  | Females | 8592 | 123 | 470 |
|  | MR CUAIBA | Total | 5987 | 118 | 503 |
|  |  | Males | 3388 | 64 | 282 |
|  |  | Females | 2599 | 54 | 221 |
|  | CAMPO GRANDE | Total | 3580 | 84 | 278 |
|  |  | Males | 1931 | 55 | 161 |
|  |  | Females | 1649 | 29 | 117 |
|  | MR RIO DE JANEIRO | Total | 56537 | 886 | 5464 |
|  |  | Males | 31276 | 442 | 3024 |
|  |  | Females | 25261 | 444 | 2440 |
|  | MR SÃO PAULO | Total | 94205 | 1805 | 9161 |
|  |  | Males | 51457 | 915 | 4982 |
|  |  | Females | 42748 | 890 | 4179 |
|  | MR CURITIBA | Total | 13553 | 154 | 975 |
|  |  | Males | 7450 | 80 | 534 |
|  |  | Females | 6103 | 74 | 441 |
|  | MR FLORIANOPOLIS | Total | 3066 | 60 | 277 |
|  |  | Males | 1685 | 29 | 150 |
|  |  | Females | 1381 | 31 | 127 |
|  | MR PORTO ALEGRE | Total | 15297 | 197 | 1346 |
|  |  | Males | 8379 | 101 | 734 |
|  |  | Females | 6918 | 96 | 612 |
| TOTAL | | **Total** | **307606** | **5458** | **26798** |
|  |  | **Males** | **169135** | **3034** | **14629** |
|  |  | **Females** | **138471** | **2424** | **12169** |
| 10-44 | MR MANAUS | Total | 22108 | 2589 | 1301 |
|  |  | Males | 12422 | 1499 | 763 |
|  |  | Females | 9686 | 1090 | 538 |
|  | MR BELEM | Total | 25029 | 3633 | 2428 |
|  |  | Males | 14015 | 2172 | 1380 |
|  |  | Females | 11014 | 1461 | 1048 |
|  | MR FORTALEZA | Total | 32008 | 5420 | 2448 |
|  |  | Males | 19291 | 3291 | 1546 |
|  |  | Females | 12717 | 2129 | 902 |
|  | MR RECIFE | Total | 40298 | 9240 | 3479 |
|  |  | Males | 24455 | 5738 | 2295 |
|  |  | Females | 15843 | 3502 | 1184 |
|  | MR SALVADOR | Total | 37334 | 8076 | 3839 |
|  |  | Males | 20669 | 4668 | 2255 |
|  |  | Females | 16665 | 3408 | 1584 |
|  | RIDE-DF | Total | 33555 | 7323 | 2000 |
|  |  | Males | 19954 | 4542 | 1209 |
|  |  | Females | 13601 | 2781 | 791 |
|  | MR CUIABA | Total | 10467 | 2113 | 773 |
|  |  | Males | 6064 | 1282 | 462 |
|  |  | Females | 4403 | 831 | 311 |
|  | CAMPO GRANDE | Total | 7015 | 1642 | 559 |
|  |  | Males | 4053 | 1027 | 356 |
|  |  | Females | 2962 | 615 | 203 |
|  | MR RIO DE JANEIRO | Total | 138142 | 28176 | 11731 |
|  |  | Males | 78072 | 16146 | 7429 |
|  |  | Females | 60070 | 1230 | 4302 |
|  | MR SÃO PAULO | Total | 198017 | 49250 | 20467 |
|  |  | Males | 119967 | 30808 | 13436 |
|  |  | Females | 78050 | 18442 | 7031 |
|  | MR CURITIBA | Total | 28499 | 5276 | 1996 |
|  |  | Males | 16948 | 3165 | 1244 |
|  |  | Females | 11551 | 2111 | 752 |
|  | MR FLORIANOPOLIS | Total | 8021 | 1593 | 669 |
|  |  | Males | 4877 | 1038 | 423 |
|  |  | Females | 3144 | 555 | 246 |
|  | MR PORTO ALEGRE | Total | 42313 | 6137 | 3327 |
|  |  | Males | 25268 | 3620 | 2131 |
|  |  | Females | 17045 | 2517 | 1196 |
| TOTAL | | **Total** | **622806** | **130468** | **55017** |
|  |  | **Males** | **366055** | **78996** | **34929** |
|  |  | **Females** | **256751** | **51472** | **20088** |
| 45-64 | MR MANAUS | Total | 41747 | 9240 | 2672 |
|  |  | Males | 24668 | 5718 | 1529 |
|  |  | Females | 17079 | 3522 | 1143 |
|  | MR BELEM | Total | 50511 | 14158 | 4965 |
|  |  | Males | 29209 | 8713 | 2942 |
|  |  | Females | 21302 | 5445 | 2023 |
|  | MR FORTALEZA | Total | 74467 | 20727 | 5631 |
|  |  | Males | 42952 | 12396 | 3131 |
|  |  | Females | 31515 | 8331 | 2500 |
|  | MR RECIFE | Total | 110912 | 41091 | 9713 |
|  |  | Males | 63915 | 24218 | 5725 |
|  |  | Females | 46997 | 16873 | 3988 |
|  | MR SALVADOR | Total | 92150 | 29943 | 7594 |
|  |  | Males | 52901 | 17701 | 4674 |
|  |  | Females | 39249 | 12242 | 2920 |
|  | RIDE-DF | Total | 74885 | 25206 | 4274 |
|  |  | Males | 44174 | 15411 | 2434 |
|  |  | Females | 30711 | 9795 | 1840 |
|  | MR CUIABA | Total | 24874 | 8392 | 1959 |
|  |  | Males | 15094 | 5162 | 1179 |
|  |  | Females | 9780 | 3230 | 780 |
|  | CAMPO GRANDE | Total | 20685 | 7615 | 1783 |
|  |  | Males | 12522 | 4843 | 1095 |
|  |  | Females | 8163 | 2772 | 688 |
|  | MR RIO DE JANEIRO | Total | 449132 | 148308 | 38036 |
|  |  | Males | 260402 | 89466 | 23005 |
|  |  | Females | 188730 | 58842 | 15031 |
|  | MR SÃO PAULO | Total | 573176 | 215037 | 54447 |
|  |  | Males | 349368 | 135163 | 34220 |
|  |  | Females | 223808 | 79874 | 20227 |
|  | MR CURITIBA | Total | 85520 | 27109 | 6715 |
|  |  | Males | 50886 | 16719 | 3684 |
|  |  | Females | 34634 | 10390 | 3031 |
|  | MR FLORIANOPOLIS | Total | 23469 | 8011 | 1734 |
|  |  | Males | 14704 | 5316 | 1111 |
|  |  | Females | 8765 | 2695 | 623 |
|  | MR PORTO ALEGRE | Total | 137941 | 38838 | 13322 |
|  |  | Males | 83110 | 23938 | 7893 |
|  |  | Females | 54831 | 14900 | 5429 |
| TOTAL | | **Total** | **1759469** | **593675** | **152845** |
|  |  | **Males** | **1043905** | **364764** | **92622** |
|  |  | **Females** | **715564** | **228911** | **60223** |
| 65-74 | MR MANAUS | Total | 29848 | 7870 | 2892 |
|  |  | Males | 17208 | 4592 | 1660 |
|  |  | Females | 12640 | 3278 | 1232 |
|  | MR BELEM | Total | 38997 | 12778 | 5523 |
|  |  | Males | 21720 | 7341 | 3105 |
|  |  | Females | 17277 | 5437 | 2418 |
|  | MR FORTALEZA | Total | 59683 | 19498 | 5953 |
|  |  | Males | 31996 | 10789 | 3453 |
|  |  | Females | 27687 | 8709 | 3204 |
|  | MR RECIFE | Total | 86519 | 34569 | 10823 |
|  |  | Males | 45011 | 18339 | 5740 |
|  |  | Females | 41508 | 16230 | 5083 |
|  | MR SALVADOR | Total | 61643 | 22370 | 6559 |
|  |  | Males | 32737 | 12245 | 3589 |
|  |  | Females | 28906 | 10125 | 2970 |
|  | RIDE-DF | Total | 53083 | 19657 | 5272 |
|  |  | Males | 29543 | 11246 | 2924 |
|  |  | Females | 23540 | 8411 | 2348 |
|  | MR CUIABA | Total | 17577 | 6511 | 2117 |
|  |  | Males | 10398 | 3899 | 1282 |
|  |  | Females | 7179 | 2612 | 835 |
|  | CAMPO GRANDE | Total | 16128 | 6337 | 2072 |
|  |  | Males | 9209 | 3672 | 1179 |
|  |  | Females | 6919 | 2665 | 893 |
|  | MR RIO DE JANEIRO | Total | 363936 | 129164 | 42249 |
|  |  | Males | 198008 | 71268 | 23887 |
|  |  | Females | 165928 | 57896 | 18362 |
|  | MR SÃO PAULO | Total | 427505 | 172758 | 54751 |
|  |  | Males | 238641 | 97125 | 31686 |
|  |  | Females | 188864 | 75633 | 23065 |
|  | MR CURITIBA | Total | 68419 | 25064 | 8355 |
|  |  | Males | 37554 | 14132 | 4227 |
|  |  | Females | 30865 | 10932 | 4128 |
|  | MR FLORIANOPOLIS | Total | 18115 | 6843 | 2051 |
|  |  | Males | 10752 | 4052 | 1298 |
|  |  | Females | 7363 | 2791 | 753 |
|  | MR PORTO ALEGRE | Total | 113247 | 37704 | 15440 |
|  |  | Males | 63033 | 20979 | 8991 |
|  |  | Females | 50214 | 16725 | 6449 |
| TOTAL | | **Total** | **1354700** | **501123** | **164057** |
|  |  | **Males** | **745810** | **279679** | **93021** |
|  |  | **Females** | **608890** | **221444** | **71740** |
| ≥ 75 | MR MANAUS | Total | 51679 | 14087 | 7355 |
|  |  | Males | 24683 | 6655 | 3415 |
|  |  | Females | 26996 | 7432 | 3940 |
|  | MR BELEM | Total | 71460 | 24505 | 15199 |
|  |  | Males | 30944 | 10580 | 6498 |
|  |  | Females | 40516 | 13925 | 8701 |
|  | MR FORTALEZA | Total | 133829 | 44519 | 23417 |
|  |  | Males | 58434 | 19856 | 9939 |
|  |  | Females | 75395 | 24663 | 13478 |
|  | MR RECIFE | Total | 156588 | 24030 | 31929 |
|  |  | Males | 63319 | 24030 | 12565 |
|  |  | Females | 93269 | 35615 | 19364 |
|  | MR SALVADOR | Total | 106927 | 39154 | 18110 |
|  |  | Males | 42774 | 15927 | 7304 |
|  |  | Females | 64153 | 23227 | 10806 |
|  | RIDE-DF | Total | 88945 | 33314 | 14801 |
|  |  | Males | 41725 | 15218 | 6992 |
|  |  | Females | 47220 | 18096 | 7809 |
|  | MR CUIABA | Total | 27865 | 10219 | 4793 |
|  |  | Males | 13863 | 4952 | 2478 |
|  |  | Females | 14002 | 5267 | 2315 |
|  | CAMPO GRANDE | Total | 29904 | 11261 | 6164 |
|  |  | Males | 14390 | 5361 | 2965 |
|  |  | Females | 15514 | 5900 | 3199 |
|  | MR RIO DE JANEIRO | Total | 675232 | 233169 | 116628 |
|  |  | Males | 272331 | 92311 | 48433 |
|  |  | Females | 402901 | 140858 | 68195 |
|  | MR SÃO PAULO | Total | 818409 | 324924 | 154310 |
|  |  | Males | 341747 | 130749 | 68706 |
|  |  | Females | 476662 | 194175 | 85604 |
|  | MR CURITIBA | Total | 116121 | 44913 | 19265 |
|  |  | Males | 50972 | 19029 | 8710 |
|  |  | Females | 65149 | 25884 | 10555 |
|  | MR FLORIANOPOLIS | Total | 35722 | 14697 | 5678 |
|  |  | Males | 15594 | 6247 | 2613 |
|  |  | Females | 20128 | 8450 | 3065 |
|  | MR PORTO ALEGRE | Total | 201577 | 75570 | 33474 |
|  |  | Males | 80842 | 28236 | 14894 |
|  |  | Females | 120735 | 47334 | 18580 |
| TOTAL | | **Total** | **2514258** | **929977** | **451123** |
|  |  | **Males** | **1051618** | **379151** | **195512** |
|  |  | **Females** | **1462640** | **550826** | **255611** |

| YEAR | MANAUS | BELEM | FORTALEZA | RECIFE | SALVADOR | RIDE-DF | CUIABA | CAMPO  GRANDE | R JANEIRO | SÃO PAULO | CURITIBA | FLORIANO-POLIS | PORTO ALEGRE | TOTAL |
| --- | --- | --- | --- | --- | --- | --- | --- | --- | --- | --- | --- | --- | --- | --- |
| 2000 | 4 | 3 | 2 | 1 | 0 | 13 | 11 | 10 | 17 | 18 | 12 | 14 | 4 | 109 |
| 2001 | 6 | 4 | 14 | 12 | 5 | 14 | 12 | 4 | 14 | 8 | 5 | 6 | 5 | 109 |
| 2002 | 5 | 7 | 0 | 3 | 5 | 15 | 19 | 13 | 16 | 18 | 11 | 9 | 5 | 126 |
| 2003 | 6 | 6 | 7 | 12 | 6 | 11 | 20 | 10 | 13 | 16 | 12 | 16 | 11 | 146 |
| 2004 | 9 | 9 | 11 | 4 | 13 | 16 | 30 | 23 | 9 | 9 | 8 | 5 | 15 | 161 |
| 2005 | 11 | 13 | 32 | 15 | 6 | 5 | 23 | 12 | 2 | 7 | 8 | 7 | 17 | 158 |
| 2006 | 4 | 13 | 7 | 18 | 12 | 6 | 7 | 11 | 11 | 13 | 26 | 26 | 16 | 170 |
| 2007 | 6 | 8 | 21 | 10 | 8 | 32 | 23 | 22 | 17 | 19 | 16 | 15 | 3 | 200 |
| 2008 | 2 | 4 | 8 | 13 | 20 | 15 | 10 | 9 | 8 | 6 | 6 | 3 | 6 | 110 |
| 2009 | 12 | 16 | 2 | 7 | 10 | 3 | 5 | 6 | 3 | 1 | 5 | 6 | 13 | 89 |
| 2010 | 15 | 14 | 25 | 13 | 4 | 28 | 30 | 20 | 12 | 11 | 4 | 5 | 5 | 186 |
| 2011 | 7 | 6 | 3 | 0 | 14 | 19 | 20 | 19 | 14 | 9 | 12 | 2 | 12 | 137 |
| 2012 | 22 | 22 | 26 | 23 | 25 | 13 | 11 | 10 | 18 | 12 | 16 | 16 | 22 | 236 |
| 2013 | 9 | 9 | 16 | 15 | 13 | 9 | 14 | 12 | 7 | 9 | 6 | 9 | 4 | 132 |
| 2014 | 10 | 6 | 3 | 2 | 6 | 5 | 2 | 12 | 24 | 24 | 12 | 9 | 4 | 119 |
| 2015 | 24 | 25 | 11 | 18 | 11 | 13 | 14 | 7 | 12 | 7 | 2 | 2 | 7 | 153 |
| 2016 | 21 | 14 | 17 | 18 | 20 | 21 | 9 | 16 | 13 | 11 | 7 | 6 | 12 | 185 |
| 2017 | 18 | 11 | 5 | 1 | 9 | 25 | 11 | 5 | 19 | 21 | 18 | 15 | 12 | 170 |
| 2018 | 15 | 5 | 2 | 14 | 10 | 9 | 4 | 13 | 8 | 15 | 20 | 12 | 7 | 134 |
| 2019 | 13 | 4 | 8 | 12 | 14 | 16 | 26 | 30 | 12 | 15 | 22 | 13 | 13 | 198 |

**Table S5.** Number of drought events (in weeks) measured by the Standardized Precipitation-Evapotranspiration Index obtained at short-term (SPEI-1) per year of study period for each Brazilian location between 2000 to 2019.

**Table S6.** Relative risks and 95% confidence intervals of weekly non-external mortality of the population separated by sex and age ranges associated with moderate drought in each Brazilian location.

| GROUPS | MANAUS | BELEM | FORTALEZA | RECIFE | SALVADOR | RIDE-DF | CUIABA | CAMPO  GRANDE | R JANEIRO | SÃO PAULO | CURITIBA | FLORIANOPOLIS | PORTO ALEGRE |
| --- | --- | --- | --- | --- | --- | --- | --- | --- | --- | --- | --- | --- | --- |
| TOTAL | 0.997  [0.977 - 1.017] | 0.986  [0.965 - 1.007] | 1.013  [0.995 - 1.031] | 0.996  [0.982 - 1.011] | 1  [0.986 - 1.015] | 1.006  [0.993 - 1.018] | 1.004  [0.985 - 1.022] | 1.01  [0.991 - 1.03] | 1.006  [0.995 - 1.018] | 1.008  [0.996 - 1.02] | 1  [0.986 -1.015] | 1.006  [0.99 - 1.022] | 0.998  [0.985 - 1.012] |
| TOTAL  0—9 | 1.011  [0.969 - 1.055] | 0.987  [0.94 - 1.036] | 1.024  [0.986 - 1.064] | 1.011  [0.977 - 1.047] | 0.986  [0.955 -1.019] | 1.003  [0.974 - 1.032] | 0.965  [0.917 - 1.016] | 1.044  [0.981 - 1.112] | 1.01  [0.992 - 1.029] | 1.021  [1.004 - 1.038] | 0.991  [0.954 - 1.029] | 1.025  [0.96 - 1.095] | 0.998  [0.964 - 1.032] |
| TOTAL  10—44 | 0.999  [0.962 - 1.036] | 0.997  [0.96 - 1.035] | 0.994  [0.964 - 1.024] | 0.98  [0.955 - 1.005] | 0.999  [0.975 -1.024] | 0.986  [0.965 - 1.008] | 1.01  [0.972 - 1.049] | 1.007  [0.963 - 1.054] | 0.999  [0.985 -1.013] | 1.005  [0.99 - 1.019] | 1.003  [0.977 - 1.03] | 1.03  [0.987 - 1.074] | 0.989  [0.968 - 1.011] |
| TOTAL  45—64 | 0.984  [0.957 - 1.013] | 0.995  [0.968 - 1.024] | 1.009  [0.986 - 1.033] | 0.992  [0.974 - 1.01] | 0.991  [0.973 - 1.01] | 1.006  [0.991 - 1.022] | 1.017  [0.99 - 1.044] | 1.018  [0.989 - 1.048] | 1.004  [0.992 -1.016] | 1.008  [0.995 - 1.021] | 0.996  [0.977 - 1.015] | 0.991  [0.966 - 1.017] | 0.994  [0.978 - 1.01] |
| TOTAL  65—74 | 1.005  [0.973 - 1.037] | 0.979  [0.949 - 1.01] | 1.037  [1.012 - 1.062] | 0.993  [0.974 - 1.013] | 1.004  [0.983 - 1.025] | 1.016  [0.999 - 1.033] | 0.993  [0.964 - 1.023] | 1.005  [0.973 - 1.038] | 1.001  [0.989 - 1.014] | 1.007  [0.994 - 1.02] | 1.006  [0.987 - 1.025] | 0.989  [0.961 - 1.017] | 1.005  [0.989 - 1.022] |
| TOTAL  ≥ 75 | 0.996  [0.971 - 1.023] | 0.978  [0.953 - 1.004] | 1.007  [0.985 - 1.029] | 1.003  [0.986 - 1.02] | 1.007  [0.989 - 1.025] | 1.006  [0.991 - 1.022] | 1.005  [0.979 - 1.032] | 1.004  [0.979 - 1.03] | 1.011  [0.999 - 1.024] | 1.007  [0.994 - 1.02] | 1  [0.983 - 1.017] | 1.017  [0.995 - 1.04] | 0.999  [0.984 - 1.013] |
| MALES | 0.991  [0.968 - 1.013] | 0.993  [0.97 - 1.016] | 1.014  [0.994 - 1.033] | 0.999  [0.983 - 1.015] | 0.996  [0.98 - 1.012] | 1.009  [0.995 - 1.022] | 1  [0.979 - 1.021] | 1.016  [0.994 - 1.039] | 1.003  [0.992 - 1.015] | 1.005  [0.993 - 1.017] | 0.994  [0.978 - 1.01] | 1.002  [0.982 - 1.022] | 0.994  [0.98 - 1.008] |
| MALES  0—9 | 0.984  [0.931 - 1.039] | 1.025  [0.966 - 1.088] | 1.042  [0.994 - 1.092] | 1.03  [0.983 - 1.078] | 0.983  [0.943 - 1.024] | 1.003  [0.967 - 1.041] | 0.986  [0.924 - 1.051] | 1.03  [0.947 - 1.121] | 1.007  [0.985 - 1.03] | 1.017  [0.997 - 1.037] | 0.967  [0.921 - 1.016] | 1.035  [0.946 - 1.132] | 1.001  [0.957 - 1.046] |
| MALES  10—44 | 0.992  [0.947 - 1.039] | 1.019  [0.972 - 1.068] | 0.981  [0.946 - 1.019] | 0.979  [0.951 - 1.009] | 0.993  [0.962 - 1.025] | 0.982  [0.956 - 1.009] | 0.995  [0.947 - 1.046] | 0.996  [0.939 - 1.056] | 0.998  [0.982 - 1.014] | 1.003  [0.987 - 1.019] | 0.991  [0.959 - 1.024] | 1.045  [0.993 - 1.1] | 0.982  [0.956 - 1.009] |
| MALES  45—64 | 0.977  [0.943 - 1.011] | 0.999  [0.965 - 1.034] | 1.015  [0.989 - 1.043] | 0.998  [0.978 - 1.019] | 0.989  [0.968 - 1.011] | 1.001  [0.983 - 1.019] | 1.021  [0.988 - 1.055] | 1.031  [0.996 - 1.068] | 0.999  [0.987 - 1.012] | 1.007  [0.993 - 1.02] | 0.988  [0.967 - 1.01] | 1.002  [0.972 - 1.033] | 0.987  [0.969 - 1.005] |
| TOTAL  65—74 | 0.995  [0.957 - 1.035] | 0.981  [0.945 - 1.018] | 1.032  [1.003 - 1.063] | 1.001  [0.977 - 1.024] | 0.999  [0.974 - 1.025] | 1.014  [0.993 - 1.036] | 0.99  [0.953 - 1.029] | 1.009  [0.968 - 1.052] | 0.999  [0.985 - 1.012] | 1.001  [0.987 - 1.015] | 1.003  [0.98 - 1.028] | 0.962  [0.927 - 0.998] | 0.998  [0.979 - 1.017] |
| TOTAL  ≥ 75 | 1.002  [0.969 - 1.036] | 0.976  [0.945 - 1.009] | 1.008  [0.981 - 1.035] | 1.001  [0.98 - 1.022] | 1.004  [0.982 - 1.027] | 1.024  [1.005 - 1.044] | 0.991  [0.958 - 1.026] | 1.011  [0.979 - 1.045] | 1.011  [0.998 - 1.025] | 1.004  [0.991 - 1.018] | 0.996  [0.975 - 1.017] | 1.012  [0.981 - 1.044] | 0.999  [0.982 - 1.017] |
| FEMALES | 1.004  [0.98 - 1.028] | 0.978  [0.955 - 1.002] | 1.012  [0.991 - 1.033] | 0.994  [0.978 - 1.01] | 1.004  [0.988 - 1.021] | 1.002  [0.988 - 1.017] | 1.008  [0.984 - 1.033] | 1.003  [0.979 - 1.028] | 1.009  [0.997 - 1.021] | 1.011  [0.998 - 1.024] | 1.007  [0.992 - 1.023] | 1.011  [0.989 - 1.032] | 1.003  [0.988 - 1.017] |
| FEMALES  0—9 | 1.044  [0.981 - 1.111] | 0.942  [0.88 - 1.009] | 1.002  [0.948 - 1.059] | 0.99  [0.944 - 1.039] | 0.991  [0.948 - 1.035] | 1.002  [0.963 - 1.043] | 0.937  [0.867 - 1.012] | 1.059  [0.966-1.162] | 1.014  [0.991 - 1.038] | 1.025  [1.004 - 1.047] | 1.019  [0.966 - 1.076] | 1.013  [0.92 - 1.115] | 0.993  [0.949 - 1.04] |
| FEMALES  10—44 | 1.007  [0.958 - 1.058] | 0.97  [0.922 - 1.022] | 1.012  [0.969 - 1.056] | 0.98  [0.944 - 1.016] | 1.007  [0.974 - 1.042] | 0.992  [0.962 - 1.024] | 1.031  [0.977 - 1.088] | 1.025  [0.956 - 1.098] | 1  [0.982 - 1.019] | 1.008  [0.99 - 1.026] | 1.021  [0.982 - 1.061] | 1.006  [0.942 - 1.074] | 0.999  [0.969 - 1.03] |
| FEMALES  45—64 | 0.996  [0.957 - 1.037] | 0.991  [0.955-1.029] | 1.001  [0.971 - 1.032] | 0.984  [0.961 - 1.007] | 0.994  [0.971 - 1.017] | 1.014  [0.993 - 1.036] | 1.011  [0.972 - 1.051] | 0.997  [0.954 - 1.041] | 1.009  [0.996 - 1.023] | 1.011  [0.996 - 1.025] | 1.007  [0.983 - 1.031] | 0.974  [0.933 - 1.016] | 1.004  [0.985 - 1.024] |
| FEMALES  65—74 | 1.017  [0.972 - 1.064] | 0.977  [0.935 - 1.02] | 1.042  [1.01 - 1.076] | 0.986  [0.962 - 1.01] | 1.009  [0.983 - 1.037] | 1.018  [0.995 - 1.042] | 0.997  [0.954 - 1.042] | 1.001  [0.954 - 1.05] | 1.004  [0.99 - 1.018] | 1.014  [0.999 - 1.029] | 1.009  [0.983 - 1.035] | 1.027  [0.984 - 1.072] | 1.015  [0.995 - 1.035] |
| FEMALES  ≥ 75 | 0.991  [0.959 - 1.024] | 0.98  [0.949 - 1.012] | 1.007  [0.982 - 1.032] | 1.004  [0.986 - 1.024] | 1.009  [0.989 - 1.03] | 0.99  [0.972 - 1.008] | 1.018  [0.983 - 1.054] | 0.998  [0.965 - 1.031] | 1.012  [0.999 - 1.025] | 1.009  [0.995 - 1.022] | 1.003  [0.984 - 1.023] | 1.021  [0.993 - 1.05] | 0.998  [0.982 - 1.015] |

**Table S7.** Relative risks and 95% confidence intervals of weekly non-external mortality of the population separated by sex and age ranges associated with severe drought in each Brazilian location.

| GROUPS | MANAUS | BELEM | FORTALEZA | RECIFE | SALVADOR | RIDE-DF | CUIABA | CAMPO  GRANDE | R JANEIRO | SÃO PAULO | CURITIBA | FLORIANOPOLIS | PORTO ALEGRE |
| --- | --- | --- | --- | --- | --- | --- | --- | --- | --- | --- | --- | --- | --- |
| TOTAL | 0.994  [0.957 - 1.032] | 0.974  [0.936 - 1.012] | 1.024  [0.99 - 1.059] | 0.993  [0.966 - 1.021] | 1  [0.974 - 1.027] | 1.011  [0.988 - 1.034] | 1.007  [0.973 - 1.041] | 1.019  [0.984 - 1.056] | 1.012  [0.99 - 1.034] | 1.014  [0.992 - 1.037] | 1  [0.974 - 1.027] | 1.011  [0.981 - 1.042] | 0.997  [0.973 - 1.022] |
| TOTAL  0—9 | 1.02  [0.942 - 1.105] | 0.976  [0.892 - 1.067] | 1.045  [0.974 - 1.122] | 1.021  [0.957 - 1.09] | 0.974  [0.917 - 1.035] | 1.005  [0.952 - 1.061] | 0.937  [0.851 - 1.03] | 1.084  [0.965 - 1.218] | 1.019  [0.986 - 1.054] | 1.039  [1.008 - 1.071] | 0.983  [0.916 - 1.054] | 1.047  [0.927 - 1.184] | 0.996  [0.934 - 1.061] |
| TOTAL  10—44 | 0.997  [0.931 - 1.068] | 0.995  [0.927 - 1.067] | 0.988  [0.934 - 1.046] | 0.963  [0.918 - 1.009] | 0.998  [0.954 - 1.045] | 0.975  [0.935 - 1.016] | 1.019  [0.949 - 1.094] | 1.014  [0.933 - 1.102] | 0.998  [0.972 - 1.024] | 1.009  [0.982 - 1.036] | 1.006  [0.958 - 1.056] | 1.056  [0.977 - 1.142] | 0.98  [0.941 - 1.021] |
| TOTAL  45—64 | 0.971  [0.921 - 1.024] | 0.992  [0.941 - 1.045] | 1.017  [0.975 - 1.062] | 0.985  [0.953 - 1.018] | 0.984  [0.951 - 1.018] | 1.012  [0.983 - 1.041] | 1.031  [0.981 - 1.084] | 1.034  [0.98 - 1.09] | 1.007  [0.985 - 1.029] | 1.015  [0.991 - 1.04] | 0.992  [0.958 - 1.027] | 0.984  [0.937 - 1.032] | 0.989  [0.96 - 1.018] |
| TOTAL  65—74 | 1.009  [0.951 - 1.07] | 0.961  [0.907 - 1.018] | 1.07  [1.023 - 1.119] | 0.988  [0.953 - 1.024] | 1.008  [0.969 - 1.048] | 1.03  [0.998 - 1.063] | 0.987  [0.934 - 1.044] | 1.01  [0.951 - 1.072] | 1.002  [0.979 - 1.026] | 1.013  [0.988 - 1.038] | 1.011  [0.975 - 1.048] | 0.979  [0.928 - 1.033] | 1.01  [0.98 - 1.041] |
| TOTAL  ≥ 75 | 0.993  [0.946 - 1.043] | 0.96  [0.914 - 1.008] | 1.013  [0.973 - 1.054] | 1.006  [0.974 - 1.038] | 1.013  [0.98 - 1.047] | 1.012  [0.983 - 1.041] | 1.009  [0.961 - 1.06] | 1.008  [0.962 - 1.057] | 1.021  [0.998 - 1.045] | 1.013  [0.989 - 1.037] | 1  [0.969 - 1.032] | 1.032  [0.991 - 1.075] | 0.997  [0.97 - 1.025] |
| MALES | 0.983  [0.942 - 1.025] | 0.987  [0.945 - 1.03] | 1.026  [0.989 - 1.063] | 0.998  [0.969 - 1.029] | 0.992  [0.963 - 1.022] | 1.016  [0.991 - 1.042] | 1  [0.962 - 1.04] | 1.03  [0.988 - 1.074] | 1.006  [0.985 - 1.028] | 1.009  [0.986 - 1.032] | 0.988  [0.959 - 1.018] | 1.003  [0.967 - 1.04] | 0.989  [0.963 - 1.015] |
| MALES  0—9 | 0.97  [0.875 - 1.074] | 1.047  [0.937 - 1.171] | 1.08  [0.989 - 1.179] | 1.056  [0.969 - 1.151] | 0.968  [0.896 - 1.046] | 1.006  [0.939 - 1.077] | 0.973  [0.863 - 1.098] | 1.057  [0.904 - 1.237] | 1.014  [0.972 - 1.057] | 1.032  [0.995 - 1.071] | 0.94  [0.858 - 1.03] | 1.066  [0.902 - 1.26] | 1.001  [0.922 - 1.088] |
| MALES  10—44 | 0.985  [0.904 - 1.073] | 1.035  [0.948 - 1.129] | 0.966  [0.901 - 1.035] | 0.962  [0.91 - 1.017] | 0.986  [0.93 - 1.047] | 0.968  [0.92 - 1.017] | 0.992  [0.904 - 1.087] | 0.992  [0.889 - 1.107] | 0.996  [0.968 - 1.025] | 1.005  [0.976 - 1.035] | 0.983  [0.926 - 1.045] | 1.086  [0.986 - 1.195] | 0.967  [0.92 - 1.016] |
| MALES  45—64 | 0.957  [0.896 - 1.021] | 0.997  [0.935 - 1.064] | 1.029  [0.979 - 1.081] | 0.996  [0.959 - 1.035] | 0.98  [0.942 - 1.021] | 1.001  [0.968 - 1.035] | 1.039  [0.977 - 1.105] | 1.059  [0.992 - 1.13] | 0.999  [0.976 - 1.023] | 1.012  [0.988 - 1.038] | 0.978  [0.939 - 1.019] | 1.004  [0.948 - 1.063] | 0.976  [0.944 - 1.009] |
| MALES  65—74 | 0.992  [0.922 - 1.067] | 0.965  [0.9 - 1.034] | 1.061  [1.005 - 1.12] | 1.001  [0.958 - 1.045] | 0.999  [0.952 - 1.047] | 1.027  [0.988 - 1.068] | 0.982  [0.915 - 1.055] | 1.017  [0.942 - 1.099] | 0.997  [0.973 - 1.023] | 1.002  [0.976 - 1.028] | 1.006  [0.963 - 1.052] | 0.93  [0.868 - 0.995] | 0.996  [0.962 - 1.031] |
| MALES  ≥ 75 | 1.004  [0.944 - 1.069] | 0.957  [0.9 - 1.017] | 1.014  [0.965 - 1.065] | 1.002  [0.963 - 1.042] | 1.008  [0.966 - 1.051] | 1.045  [1.009 - 1.084] | 0.984  [0.924 - 1.048] | 1.021  [0.96 - 1.086] | 1.021  [0.996 - 1.047] | 1.008  [0.983 - 1.033] | 0.992  [0.954 - 1.032] | 1.022  [0.965 - 1.083] | 0.999  [0.967 - 1.032] |
| FEMALES | 1.007  [0.963 - 1.053] | 0.96  [0.917 - 1.004] | 1.022  [0.984 - 1.062] | 0.989  [0.959 - 1.019] | 1.008  [0.978 - 1.039] | 1.004  [0.978 - 1.031] | 1.015  [0.971 - 1.061] | 1.006  [0.961 - 1.053] | 1.017  [0.995 - 1.04] | 1.02  [0.996 - 1.044] | 1.014  [0.984 - 1.044] | 1.02  [0.98 - 1.061] | 1.005  [0.979 - 1.033] |
| FEMALES  0—9 | 1.083  [0.965 - 1.215] | 0.896  [0.789 - 1.017] | 1.004  [0.906 - 1.112] | 0.982  [0.899 - 1.073] | 0.983  [0.905 - 1.067] | 1.004  [0.932 - 1.082] | 0.885  [0.766 - 1.023] | 1.113  [0.937 - 1.322] | 1.026  [0.983 - 1.071] | 1.048  [1.007 - 1.09] | 1.036  [0.937 - 1.145] | 1.024  [0.856 - 1.225] | 0.988  [0.907 - 1.076] |
| FEMALES  10—44 | 1.013  [0.924 - 1.111] | 0.945  [0.859 - 1.041] | 1.022  [0.944 - 1.106] | 0.962  [0.899 - 1.03] | 1.014  [0.952 - 1.08] | 0.986  [0.93 - 1.045] | 1.058  [0.957 - 1.17] | 1.047  [0.92 - 1.191] | 1  [0.967 - 1.035] | 1.015  [0.981 - 1.049] | 1.04  [0.967 - 1.117] | 1.01  [0.894 - 1.142] | 0.998  [0.943 - 1.057] |
| FEMALES  45—64 | 0.992  [0.921 - 1.069] | 0.984  [0.918 - 1.054] | 1.002  [0.947 - 1.06] | 0.97  [0.929 - 1.013] | 0.988  [0.946 - 1.032] | 1.027  [0.987 - 1.068] | 1.02  [0.948 - 1.096] | 0.994  [0.916 - 1.078] | 1.018  [0.993 - 1.043] | 1.02  [0.993 - 1.048] | 1.013  [0.968 - 1.059] | 0.951  [0.879 - 1.029] | 1.008  [0.972 - 1.046] |
| FEMALES  65—74 | 1.031  [0.948 - 1.122] | 0.957  [0.883 - 1.037] | 1.08  [1.018 - 1.146] | 0.974  [0.931 - 1.019] | 1.018  [0.968 - 1.069] | 1.034  [0.99 - 1.08] | 0.995  [0.917 - 1.08] | 1.001  [0.916 - 1.094] | 1.008  [0.982 - 1.034] | 1.026  [0.999 - 1.054] | 1.016  [0.969 - 1.065] | 1.051  [0.971 - 1.138] | 1.028  [0.99 - 1.067] |
| FEMALES  ≥ 75 | 0.983  [0.926 - 1.045] | 0.962  [0.907 - 1.022] | 1.012  [0.967 - 1.06] | 1.008  [0.973 - 1.044] | 1.017  [0.979 - 1.056] | 0.981  [0.948 - 1.016] | 1.034  [0.969 - 1.104] | 0.996  [0.936 - 1.059] | 1.022  [0.997 - 1.046] | 1.016  [0.991 - 1.041] | 1.006  [0.97 - 1.043] | 1.04  [0.988 - 1.094] | 0.996  [0.966 - 1.027] |

**Table S8.** Relative risks and 95% confidence intervals of weekly non-external mortality of the population separated by sex and age ranges associated with extreme drought in each Brazilian location.

| GROUPS | MANAUS | BELEM | FORTALEZA | RECIFE | SALVADOR | RIDE-DF | CUIABA | CAMPO  GRANDE | RJANEIRO | SÃO PAULO | CURITIBA | FLORIANOPOLIS | PORTO ALEGRE |
| --- | --- | --- | --- | --- | --- | --- | --- | --- | --- | --- | --- | --- | --- |
| TOTAL | 0.989  [0.923 - 1.059] | 0.952  [0.885 - 1.023] | 1.045  [0.982 - 1.112] | 0.988  [0.938 - 1.04] | 1  [0.952 - 1.051] | 1.02  [0.978 - 1.064] | 1.012  [0.951 - 1.077] | 1.036  [0.97 - 1.106] | 1.022  [0.982 - 1.063] | 1.027  [0.985 - 1.07] | 1.001  [0.952 - 1.051] | 1.02  [0.965 - 1.079] | 0.994  [0.95 - 1.041] |
| TOTAL  0—9 | 1.038  [0.896 - 1.203] | 0.955  [0.81 - 1.128] | 1.086  [0.953 - 1.236] | 1.039  [0.922 - 1.172] | 0.953  [0.853 - 1.065] | 1.01  [0.914 - 1.116] | 0.886  [0.742 - 1.057] | 1.161  [0.936 - 1.44] | 1.036  [0.974 - 1.102] | 1.074  [1.015 - 1.136] | 0.969  [0.851 - 1.103] | 1.089  [0.869 - 1.366] | 0.992  [0.882 - 1.115] |
| TOTAL  10—44 | 0.995  [0.877 - 1.129] | 0.99  [0.87 - 1.127] | 0.978  [0.881 - 1.086] | 0.932  [0.854 - 1.016] | 0.997  [0.917 - 1.085] | 0.954  [0.884 - 1.029] | 1.035  [0.907 - 1.18] | 1.026  [0.879 - 1.198] | 0.996  [0.949 - 1.045] | 1.016  [0.967 - 1.068] | 1.011  [0.925 - 1.106] | 1.106  [0.957 - 1.278] | 0.963  [0.893 - 1.039] |
| TOTAL  45—64 | 0.947  [0.859 - 1.045] | 0.984  [0.894 - 1.084] | 1.032  [0.954 - 1.117] | 0.973  [0.915 - 1.034] | 0.97  [0.912 - 1.033] | 1.022  [0.969 - 1.077] | 1.058  [0.965 - 1.161] | 1.063  [0.963 - 1.174] | 1.013  [0.972 - 1.055] | 1.029  [0.984 - 1.075] | 0.986  [0.924 - 1.051] | 0.97  [0.887 - 1.061] | 0.979  [0.927 - 1.034] |
| TOTAL  65—74 | 1.016  [0.911 - 1.133] | 0.929  [0.835 - 1.034] | 1.133  [1.043 - 1.232] | 0.978  [0.914 - 1.046] | 1.014  [0.943 - 1.09] | 1.056  [0.996 - 1.12] | 0.977  [0.881 - 1.083] | 1.018  [0.911 - 1.138] | 1.004  [0.961 - 1.048] | 1.024  [0.978 - 1.071] | 1.02  [0.955 - 1.09] | 0.961  [0.871 - 1.061] | 1.018  [0.963 - 1.077] |
| TOTAL  ≥ 75 | 0.988  [0.903 - 1.081] | 0.927  [0.847 - 1.015] | 1.024  [0.951 - 1.103] | 1.01  [0.952 - 1.072] | 1.024  [0.964 - 1.088] | 1.022  [0.969 - 1.078] | 1.017  [0.929 - 1.113] | 1.015  [0.931 - 1.107] | 1.04  [0.996 - 1.085] | 1.023  [0.98 - 1.069] | 1  [0.943 - 1.06] | 1.061  [0.984 - 1.144] | 0.995  [0.946 - 1.047] |
| MALES | 0.968  [0.896 - 1.047] | 0.975  [0.901 - 1.056] | 1.048  [0.98 - 1.12] | 0.997  [0.943 - 1.054] | 0.986  [0.933 - 1.042] | 1.03  [0.983 - 1.079] | 1  [0.931 - 1.074] | 1.057  [0.978 - 1.141] | 1.012  [0.972 - 1.053] | 1.016  [0.975 - 1.06] | 0.979  [0.926 - 1.034] | 1.006  [0.941 - 1.076] | 0.98  [0.933 - 1.028] |
| MALES  0--9 | 0.944  [0.782 - 1.141] | 1.089  [0.886 - 1.338] | 1.153  [0.981 - 1.356] | 1.106  [0.943 - 1.297] | 0.941  [0.816 - 1.086] | 1.011  [0.89 - 1.148] | 0.951  [0.761 - 1.188] | 1.109  [0.829 - 1.483] | 1.025  [0.95 - 1.107] | 1.06  [0.991 - 1.135] | 0.892  [0.753 - 1.055] | 1.126  [0.827 - 1.535] | 1.003  [0.86 - 1.169] |
| MALES  10—44 | 0.972  [0.83 - 1.14] | 1.066  [0.907 - 1.252] | 0.937  [0.825 - 1.065] | 0.931  [0.84 - 1.031] | 0.975  [0.874 - 1.088] | 0.941  [0.857 - 1.032] | 0.985  [0.83 - 1.168] | 0.985  [0.804 - 1.206] | 0.993  [0.941 - 1.047] | 1.009  [0.956 - 1.066] | 0.969  [0.867 - 1.084] | 1.164  [0.975 - 1.39] | 0.94  [0.858 - 1.03] |
| MALES  45—64 | 0.922  [0.817 - 1.04] | 0.995  [0.884 - 1.121] | 1.054  [0.961 - 1.156] | 0.993  [0.926 - 1.065] | 0.964  [0.895 - 1.039] | 1.002  [0.941 - 1.066] | 1.073  [0.958 - 1.203] | 1.111  [0.985 - 1.253] | 0.998  [0.956 - 1.043] | 1.023  [0.977 - 1.071] | 0.96  [0.89 - 1.035] | 1.007  [0.907 - 1.119] | 0.955  [0.898 - 1.016] |
| MALES  65—74 | 0.985  [0.86 - 1.127] | 0.935  [0.823 - 1.064] | 1.116  [1.01 - 1.234] | 1.002  [0.924 - 1.086] | 0.998  [0.914 - 1.089] | 1.05  [0.977 - 1.129] | 0.967  [0.848 - 1.104] | 1.032  [0.896 - 1.19] | 0.995  [0.95 - 1.042] | 1.004  [0.956 - 1.053] | 1.012  [0.932 - 1.099] | 0.874  [0.77 - 0.992] | 0.992  [0.93 - 1.059] |
| MALES  ≥ 75 | 1.008  [0.898 - 1.131] | 0.921  [0.823 - 1.031] | 1.026  [0.937 - 1.124] | 1.003  [0.932 - 1.079] | 1.014  [0.939 - 1.095] | 1.086  [1.016 - 1.16] | 0.971  [0.864 - 1.091] | 1.04  [0.928 - 1.165] | 1.039  [0.992 - 1.088] | 1.014  [0.968 - 1.063] | 0.986  [0.916 - 1.061] | 1.041  [0.935 - 1.16] | 0.998  [0.941 - 1.059] |
| FEMALES | 1.013  [0.932 - 1.1] | 0.927  [0.852 - 1.008] | 1.042  [0.971 - 1.118] | 0.979  [0.926 - 1.035] | 1.015  [0.96 - 1.072] | 1.008  [0.96 - 1.059] | 1.028  [0.947 - 1.117] | 1.012  [0.93 - 1.101] | 1.032  [0.991 - 1.075] | 1.038  [0.993 - 1.084] | 1.025  [0.971 - 1.082] | 1.037  [0.964 - 1.116] | 1.01  [0.961 - 1.061] |
| FEMALES  0—9 | 1.159  [0.937 - 1.435] | 0.815  [0.644 - 1.032] | 1.007  [0.834 - 1.217] | 0.967  [0.821 - 1.139] | 0.968  [0.832 - 1.127] | 1.008  [0.878 - 1.157] | 0.798  [0.611 - 1.043] | 1.219  [0.886 - 1.676] | 1.049  [0.969 - 1.135] | 1.09  [1.013 - 1.172] | 1.068  [0.887 - 1.286] | 1.045  [0.75 - 1.456] | 0.978  [0.834 - 1.146] |
| FEMALES  10—44 | 1.024  [0.863 - 1.215] | 0.901  [0.755 - 1.076] | 1.04  [0.899 - 1.204] | 0.932  [0.822 - 1.057] | 1.026  [0.913 - 1.152] | 0.974  [0.874 - 1.085] | 1.11  [0.922 - 1.337] | 1.088  [0.858 - 1.381] | 1  [0.94 - 1.065] | 1.027  [0.965 - 1.093] | 1.075  [0.94 - 1.228] | 1.019  [0.813 - 1.278] | 0.997  [0.896 - 1.108] |
| FEMALES  45—64 | 0.986  [0.859 - 1.132] | 0.97  [0.853 - 1.103] | 1.003  [0.903 - 1.114] | 0.945  [0.872 - 1.024] | 0.979  [0.903 - 1.06] | 1.05  [0.976 - 1.13] | 1.037  [0.907 - 1.185] | 0.989  [0.851 - 1.15] | 1.033  [0.986 - 1.081] | 1.037  [0.986 - 1.09] | 1.024  [0.942 - 1.112] | 0.912  [0.788 - 1.055] | 1.015  [0.948 - 1.086] |
| FEMALES  65—74 | 1.059  [0.905 - 1.238] | 0.922  [0.795 - 1.069] | 1.153  [1.034 - 1.286] | 0.952  [0.875 - 1.036] | 1.033  [0.942 - 1.132] | 1.064  [0.981 - 1.153] | 0.991  [0.852 - 1.154] | 1.002  [0.851 - 1.182] | 1.014  [0.967 - 1.064] | 1.049  [0.998 - 1.103] | 1.03  [0.944 - 1.124] | 1.096  [0.947 - 1.27] | 1.052  [0.982 - 1.127] |
| FEMALES  ≥ 75 | 0.97  [0.867 - 1.085] | 0.932  [0.834 - 1.04] | 1.023  [0.939 - 1.113] | 1.015  [0.951 - 1.084] | 1.031  [0.962 - 1.105] | 0.966  [0.906 - 1.03] | 1.064  [0.943 - 1.2] | 0.992  [0.884 - 1.112] | 1.04  [0.995 - 1.087] | 1.03  [0.984 - 1.078] | 1.01  [0.945 - 1.08] | 1.075  [0.977 - 1.181] | 0.993  [0.938 - 1.051] |

**Table S9.** Relative risks and 95% confidence intervals of weekly specific cause mortality of the population separated by sex and age ranges associated with moderate (M), severe (S), extreme (E) drought in each Brazilian location.

| CIRCULATORY MORTALITY | | | | | | | | | | | | | | |
| --- | --- | --- | --- | --- | --- | --- | --- | --- | --- | --- | --- | --- | --- | --- |
| GROUPS | | MANAUS | BELEM | FORTALEZA | RECIFE | SALVADOR | RIDE-DF | CUIABA | CAMPO GRANDE | R JANEIRO | SÃO PAULO | CURITIBA | FLORIANÓPOLIS | PORTO ALEGRE |
| TOTAL | M | 0.999  [0.97 - 1.03] | 0.993  [0.966 - 1.021] | 1.015  [0.993 - 1.037] | 0.994  [0.977 - 1.011] | 0.979  [0.959 - 1] | 1.015  [1 - 1.031] | 0.995  0.969 - 1.022] | 1.009  [0.983 - 1.035] | 1.002  [0.99 - 1.014] | 1.007  [0.994 - 1.02] | 1.005  [0.987 - 1.022] | 0.995  [0.972 - 1.02] | 1.002  [0.987 - 1.017] |
|  | S | 0.999  [0.944 - 1.056] | 0.988  [0.938 - 1.04] | 1.028  [0.987 - 1.071] | 0.988  [0.957 - 1.02] | 0.962  [0.925 - 1] | 1.028  [1.000 - 1.058] | 0.991  [0.943 - 1.041] | 1.017  [0.969 - 1.067] | 1.004  [0.982 - 1.027] | 1.013  [0.99 - 1.037] | 1.009  [0.976 - 1.042] | 0.991  [0.948 - 1.037] | 1.003  [0.975 - 1.032] |
|  | E | 0.998  [0.899 - 1.107] | 0.977  [0.889 - 1.075] | 1.053  [0.977 - 1.134] | 0.978  [0.922 - 1.037] | 0.93  [0.866 - 1] | 1.053  [0.999 - 1.11] | 0.983  [0.897 - 1.078] | 1.031  [0.943 - 1.127] | 1.008  [0.967 - 1.051] | 1.025  [0.981 - 1.07] | 1.016  [0.957 - 1.079] | 0.984  [0.906 - 1.069] | 1.006  [0.954 - 1.061] |
| MALES | M | 1.000  [0.963 - 1.039] | 0.997  [0.963 - 1.033] | 1.014  [0.989 - 1.04] | 1.002  [0.982 - 1.022] | 0.951  [0.924 - 0.978] | 1.023  [1.005 - 1.042] | 0.991  [0.958 - 1.024] | 1.014  [0.982 - 1.047] | 0.996  [0.984 - 1.01] | 1.003  [0.99 - 1.016] | 0.989  [0.968 - 1.01] | 0.977  [0.948 - 1.007] | 0.998  [0.98 - 1.016] |
|  | S | 1 .000  [0.932 - 1.074] | 0.995  [0.932 - 1.061] | 1.027  [0.98 - 1.076] | 1.003  [0.967 - 1.041] | 0.91  [0.864 - 0.959] | 1.044  [1.01 - 1.079] | 0.982  [0.923 - 1.046] | 1.026  [0.968 - 1.089] | 0.993  [0.97 - 1.018] | 1.005  [0.981 - 1.03] | 0.979  [0.941 - 1.019] | 0.958  [0.906 - 1.014] | 0.997  [0.964 - 1.031] |
|  | E | 1.001  [0.878 - 1.141] | 0.99  [0.879 - 1.116] | 1.051  [0.964 - 1.145] | 1.006  [0.939 - 1.077] | 0.84  [0.763 - 0.926] | 1.083  [1.019 - 1.151] | 0.968  [0.862 - 1.086] | 1.049  [0.941 - 1.17] | 0.988  [0.945 - 1.033] | 1.01  [0.966 - 1.056] | 0.962  [0.894 - 1.036] | 0.924  [0.833 - 1.026] | 0.994  [0.934 - 1.058] |
| FEMALES | M | 0.998  [0.959 - 1.04] | 0.989  [0.954 - 1.025] | 1.016  [0.988 - 1.044] | 0.986  [0.966 - 1.006] | 1.012  [0.99 - 1.035] | 1.005  [0.985 - 1.026] | 1.001  [0.965 - 1.037] | 1.002  [0.966 - 1.04] | 1.008  [0.995 - 1.021] | 1.012  [0.998 - 1.025] | 1.022  [1 - 1.044] | 1.016  [0.982 - 1.051] | 1.005  [0.988 - 1.023] |
|  | S | 0.997  [0.925 - 1.075] | 0.98  [0.917 - 1.047] | 1.029  [0.978 - 1.083] | 0.973  [0.937 - 1.011] | 1.022  [0.981 - 1.065] | 1.01  [0.972 - 1.049] | 1.001  [0.937 - 1.07] | 1.004  [0.938 - 1.075] | 1.015  [0.991 - 1.04] | 1.022  [0.996 - 1.048] | 1.04  [1 - 1.083] | 1.03  [0.967 - 1.096] | 1.01  [0.978 - 1.043] |
|  | E | 0.995  [0.865 - 1.143] | 0.963  [0.851 - 1.089] | 1.055  [0.959 - 1.159] | 0.951  [0.886 - 1.021] | 1.041  [0.964 - 1.124] | 1.018  [0.948 - 1.093] | 1.003  [0.886 - 1.134] | 1.008  [0.889 - 1.144] | 1.029  [0.984 - 1.075] | 1.04  [0.993 - 1.09] | 1.076  [0.999 - 1.159] | 1.055  [0.94 - 1.185] | 1.018  [0.959 - 1.081] |
| RESPIRATORY MORTALITY | | | | | | | | | | | | | | |
| GROUPS | | MANAUS | BELEM | FORTALEZA | RECIFE | SALVADOR | RIDE-DF | CUIABA | CAMPO GRANDE | R JANEIRO | SÃO PAULO | CURITIBA | FLORIANÓPOLIS | PORTO ALEGRE |
| TOTAL | M | 0.976  [0.933 - 1.021] | 0.946  [0.91 - 0.984] | 1.02  [0.987 - 1.054] | 0.997  [0.973 - 1.022] | 1.005  [0.98 - 1.031] | 1.006  [0.982 - 1.03] | 1.032  [0.993 - 1.073] | 1.009  [0.979 - 1.04] | 1.01  [0.995 - 1.025] | 1.02  [1.005 - 1.036] | 0.994  [0.969 - 1.02] | 1.005  [0.968 - 1.044] | 0.991  [0.971 - 1.012] |
|  | S | 0.956  [0.88 - 1.039] | 0.902  [0.839 - 0.97] | 1.037  [0.976 - 1.102] | 0.995  [0.951 - 1.041] | 1.01  [0.963 - 1.059] | 1.011  [0.967 - 1.057] | 1.061  [0.987 - 1.141] | 1.017  [0.961 - 1.076] | 1.019  [0.99 - 1.048] | 1.038  [1.009 - 1.067] | 0.989  [0.943 - 1.038] | 1.01  [0.942 - 1.083] | 0.984  [0.947 - 1.023] |
|  | E | 0.92  [0.789 - 1.074] | 0.827  [0.723 - 0.945] | 1.07  [0.956 - 1.197] | 0.99  [0.911 - 1.076] | 1.018  [0.932 - 1.112] | 1.021  [0.941 - 1.108] | 1.116  [0.975 - 1.276] | 1.031  [0.929 - 1.145] | 1.035  [0.982 - 1.09] | 1.071  [1.017 - 1.128] | 0.98  [0.896 - 1.072] | 1.018  [0.895 - 1.159] | 0.971  [0.904 - 1.043] |
| MALES | M | 0.961  [0.906 - 1.018] | 0.935  [0.891 - 0.982] | 1.008  [0.969 - 1.049] | 0.999  [0.969 - 1.03] | 0.99  [0.958 - 1.024] | 1.001  [0.971 - 1.03] | 1.019  [0.967 - 1.073] | 1.013  [0.979 - 1.048] | 1.008  [0.992 - 1.025] | 1.016  [1 - 1.032] | 0.984  [0.952 - 1.017] | 0.993  [0.943 - 1.045] | 0.985  [0.961 - 1.01] |
|  | S | 0.928  [0.833 - 1.034] | 0.883  [0.806 - 0.967] | 1.016  [0.943 - 1.093] | 0.999  [0.944 - 1.057] | 0.982  [0.923 - 1.045] | 1.001  [0.947 - 1.057] | 1.035  [0.94 - 1.14] | 1.024  [0.962 - 1.091] | 1.016  [0.986 - 1.047] | 1.03  [1 - 1.061] | 0.97  [0.912 - 1.032] | 0.987  [0.897 - 1.086] | 0.972  [0.928 - 1.018] |
|  | E | 0.871  [0.713 - 1.064] | 0.794  [0.672 - 0.939] | 1.029  [0.898 - 1.18] | 0.998  [0.899 - 1.109] | 0.967  [0.862 - 1.084] | 1.002  [0.905 - 1.109] | 1.065  [0.891 - 1.274] | 1.045  [0.931 - 1.174] | 1.03  [0.974 - 1.089] | 1.057  [1.001 - 1.116] | 0.946  [0.844 - 1.061] | 0.975  [0.817 - 1.164] | 0.949  [0.871 - 1.033] |
| FEMALES | M | 0.992  [0.932 - 1.055] | 0.957  [0.91 - 1.006] | 1.03  [0.989 - 1.074] | 0.995  [0.967 - 1.024] | 1.02  [0.987 - 1.054] | 1.012  [0.98 - 1.044] | 1.051  [0.994 - 1.111] | 1.000  [0.945 - 1.057] | 1.011  [0.994 - 1.029] | 1.024  [1.007 - 1.042] | 1.004  [0.972 - 1.038] | 1.019  [0.967 - 1.074] | 1.051  [0.994 - 1.111] |
|  | S | 0.984  [0.878 - 1.104] | 0.922  [0.84 - 1.012] | 1.057  [0.98 - 1.141] | 0.991  [0.94 - 1.045] | 1.037  [0.975 - 1.102] | 1.022  [0.963 - 1.084] | 1.097  [0.989 - 1.217] | 0.999  [0.901 - 1.108] | 1.021  [0.989 - 1.055] | 1.046  [1.013 - 1.08] | 1.008  [0.948 - 1.072] | 1.035  [0.939 - 1.141] | 1.097  [0.989 - 1.217] |
|  | E | 0.971  [0.785 - 1.201] | 0.86  [0.724 - 1.021] | 1.109  [0.963 - 1.277] | 0.983  [0.891 - 1.084] | 1.069  [0.955 - 1.197] | 1.041  [0.933 - 1.161] | 1.186  [0.979 - 1.437] | 0.999  [0.824 - 1.21] | 1.04  [0.979 - 1.104] | 1.087  [1.025 - 1.152] | 1.015  [0.906 - 1.137] | 1.066  [0.891 - 1.277] | 1.186  [0.979 - 1.437] |


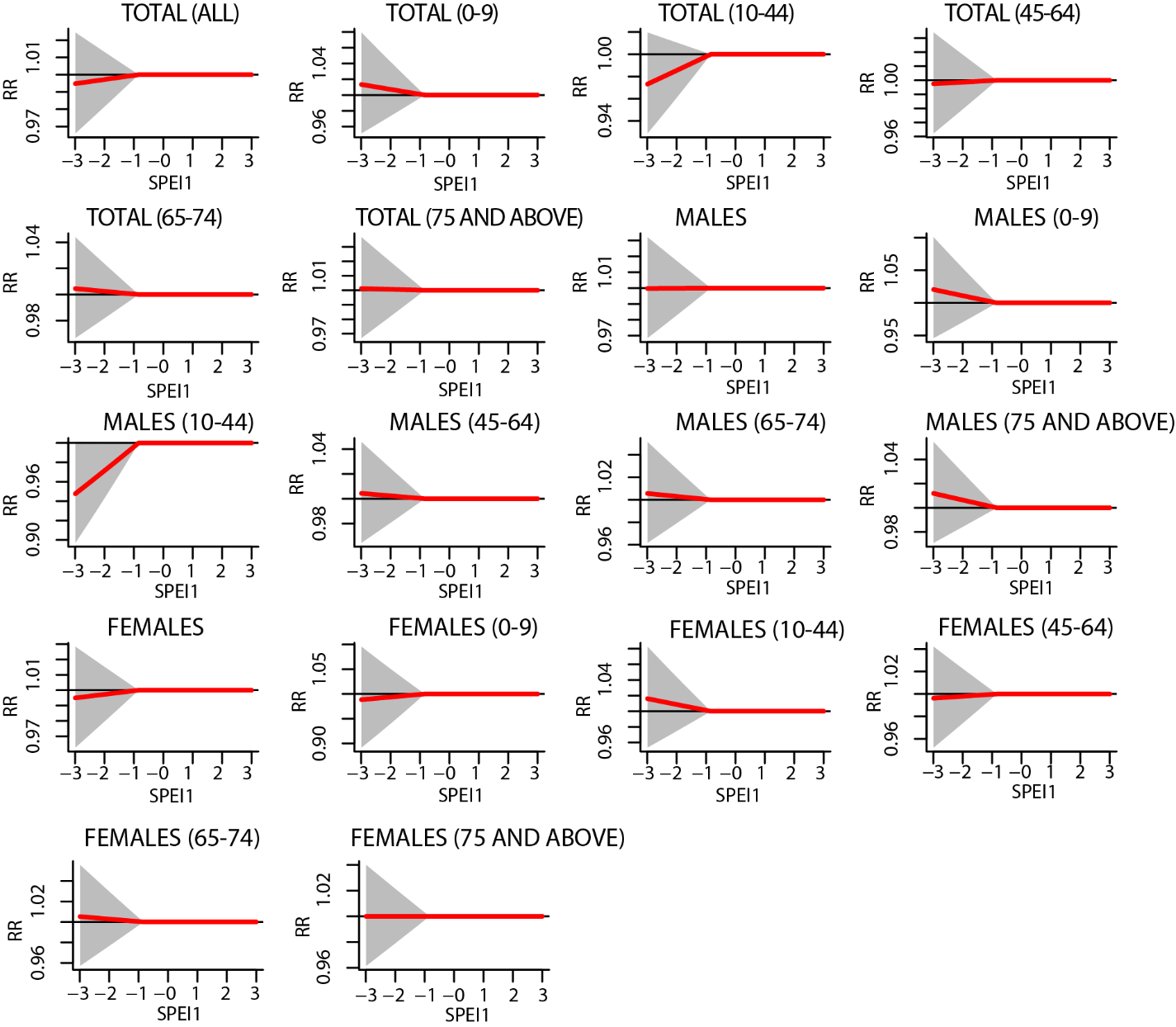


**Figure S4.** The overall association between drought and non-external mortality expressed as relative risks (RRs) (red) and 95% confidence intervals (grey area) when the lag dimension was extended to 3 weeks in the different population groups.
